# Research on the coordinated development between land urbanization and population urbanization in Shaanxi Province, China

**DOI:** 10.1038/s41598-024-58625-3

**Published:** 2024-04-04

**Authors:** Zhao Hangli, Ai Xinnan

**Affiliations:** School of Management, Northwest University of Political Science and Law, Xi’an, 710063 China

**Keywords:** Land urbanization and population urbanization, Coordinated development, Comprehensive evaluation, Coupling coordination degree, Analytic Network Process (ANP), Environmental impact, Socioeconomic scenarios, Sustainability, Environmental economics, Urban ecology

## Abstract

The coordinated development of land urbanization and population urbanization is crucial for the advancement of new urbanization. The study applied the entropy weight method and coupling coordination degree model, taking Shaanxi—a province in China characterized by a moderate pace of economic development and volume, along with distinct geographic and demographic features within its region—as the subject. It assessed the coordination conditions of these two types of urbanization from both macro and micro scales during the years 2010–2022. Utilizing the Analytic Network Process (ANP), the study ranked and analyzed the causes of issues stemming from uneven development, thus connecting a crucial link from theoretical analysis to decision-making implementation. The results showed that: (1) The province's land urbanization index was between 0.075 and 0.203, whereas the population urbanization index ranged from 0.221 to 0.408, with the development of the former significantly lagging behind the latter. (2) The coupling degree between land and population urbanization ranged from 0.835 to 0.854, with a coordination degree between 0.148 and 0.306. This indicated that a close connection had been formed between the two, yet a benign coupling relationship had not been established, displaying a spatial distribution characterized by "high in the middle, low in the north and south". (3) The limitation on further urban expansion was identified as the primary issue to be addressed (with a weight of 0.324), followed by insufficient infrastructure (with a weight of 0.261). The extent of ecological environmental damage was comparatively lower (with a weight of 0.225), and the degree of social injustice was the lowest (with a weight of 0.191). Therefore, to alleviate the problems associated with the imbalanced development between land urbanization and population urbanization, measures such as optimizing land spatial layout, enhancing urban ecological service functions, and strengthening the central cities' radiating effect should be implemented.

## Introduction

Urbanization is a microcosm of the social and economic development process. The concept of urbanization was introduced into China in the 1970s. Zhou’s translation into Chinese for the first time was widely recognized and accepted by Chinese scholars^1^. With the sustained development and improvement of market economy and economic system, China’s urbanization process is accelerating. From 1983 to 2005, China’s urbanization rate increased from 21.62 to 42.99% in just 20-odd years. For the same level of development, it took nearly 120 years for Great Britain, 100 years for France, 80 years for Germany and 40 years for the United States^2^. The rapid urbanization process inevitably leads to a series of problems in economic, social, and environmental aspects. To mitigate the impacts of rapid urbanization, Chinese government have introduced the concept of "new urbanization." This approach centers on the well-being of the population, balancing industrial development, the free flow of factors, innovations in institutional policies and mechanisms, and the construction of ecological civilization across multiple dimensions of urbanization^3^

In the process of constructing new urbanization, the population is the main driver of urbanization development, while land provides the space for urbanization's expansion. Population urbanization is the core part of the "new urbanization". Scholars have explored the connotation of population urbanization from different perspectives and disciplinary backgrounds, yet there is no consensus in the academic community. Some scholars believed that population urbanization mainly referred to the growth of urban population^4–6^, but the traditional "urbanization rate" measured by the proportion of urban population was not sufficient to fully reflect the socio-economic development process of the vast majority of developing countries, nor was it necessarily the case that higher was better. Some scholars also believed that population urbanization not only included the continuous increase in the urban population ratio but also encompassed the improvement in the quality of urban residents' lives^7–9^. In line with the connotation of "new urbanization," this paper posited that population urbanization mainly referred to the continuous increase in the proportion of urban population, the ongoing transition from agriculture to non-agriculture, the continuous improvement in residents' living quality, and the constant modernization of rural lifestyles. Land urbanization is the most important aspect of China's "new urbanization" process. Some scholars believed that the connotation of land urbanization could be measured by the proportion of the urban built-up area to the total area^4,5^. Some scholars also believed that land urbanization was not only reflected in the expansion of urban built-up areas but also in the improvement of land use efficiency^10,11^. Based on scholars' viewpoints, this paper believed that land urbanization was mainly reflected in three aspects: the rationalization of land structure, the increase in the level of land input, and the growth of land output.

As a typical region, Shaanxi Province faced two main issues in the construction of new urbanization: spatial congestion and land idleness^12^. Firstly, spatial congestion was primarily evident in the high population density of central cities and central urban areas, where urban public infrastructure operated over its capacity. Such areas, due to mature land development and a complete infrastructure and public service system, attracted population spontaneously to the center, causing spatial congestion in these areas, which in turn led to secondary issues such as environmental degradation. Secondly, land idleness was manifested in the idleness and inefficient use of land in peripheral cities and suburban areas. These areas, due to their relative distance from central urban areas and insufficient infrastructure, suffered from poor population agglomeration, leading to inefficient land use and wastage, which restricted further urban expansion.

In Shaanxi Province, the issues of spatial congestion and land vacancy existed side by side, reflected in various real-world problems such as traffic congestion, smoggy weather, environmental degradation, and housing pressure, among others. These could be explained through discussions related to the relationship between population urbanization and land urbanization. This implied that an analysis of the relationship between population urbanization and land urbanization in Shaanxi Province was necessary, which could alleviate or resolve some of these practical issues, having significant real-world importance. Furthermore, most of the existing literature was based on national or regional data, with few studies utilizing provincial panel data, and research on the urbanization of population and land in Shaanxi Province was also limited. Based on this, the paper selected Shaanxi Province, which had a relatively moderate economic development speed and volume and distinct geographical and demographic characteristics within the region, as the evidence subject. It discussed the coordinated development status and impact of population urbanization and land urbanization from both macro and micro perspectives, contributing to the enrichment of the theoretical framework of current research.

Therefore, this paper explored the coordinated development status of population urbanization and land urbanization between 2010 and 2022 and conducted an in-depth analysis of its impact, with the aim of providing a scientific basis for decision-making related to the sustainable development of new urbanization in China in the new era, and offering reference for the solution of similar issues in developing countries worldwide.

## Literature review

In existing research, many scholars have studied "urbanization" as a comprehensive system, and the exploration of factors influencing urbanization development has remained a hot topic in current research. Factors such as growth in oil income, domestic migration, rational urban planning, foreign direct investment, and trade have all been considered by scholars to be positive factors that effectively promoted urbanization development^13–15^. Paul believed that legal constraints, increased demand for ecotourism, and enhanced environmental awareness were favorable for the sustainable development of urbanization^16^. Jiang’s research found that urbanization development could further narrow the gaps in fiscal investment, labor productivity, and quality of life between urban and rural areas. It was recommended to formulate relevant policy measures tailored to local conditions to promote sustainable development of the rural economy^17^. And Li believed that rapid urbanization caused by changes in land use was one of the reasons for the fragility of land institutions. Additionally, excessive urbanization was also not conducive to the sustainable development of urban ecosystems^18^.

Furthermore, numerous scholars have extensively studied the relationship between ecological environment, biological population factors, and economic development in the process of urbanization. The majority of their conclusions tend to indicate that urbanization has negative impacts on ecological environment and biological populations, while simultaneously playing a promoting role in social and economic development^19–24^. However, there were also some scholars who held different opinions. For example, Faisal believed that when urbanization reached a certain threshold, it would instead reduce environmental pollution^25^. Atisa proposed that there was a very strong leverage point between urbanization and species diversity, and discovering and utilizing this leverage point could accumulate the expected ecological restoration effects^26^.

Some scholars explored the relationship between population urbanization and land urbanization from a theoretical perspective^27^. Yan argued that population urbanization could promote the development of land urbanization. However, relying solely on land urbanization was not effective in driving and promoting population urbanization^28^. Li conducted a quantitative study on the dynamic effects of urbanization systems in Beijing, and the results showed that the expansion of urban construction land can sustainably support the healthy development of population and economic urbanization. However, there are still issues such as insufficient support and weak interaction among different urbanization systems, which necessitates the promotion of coordinated development among them^29^.

Population urbanization and land urbanization, as the core and key aspects of the "new urbanization", have attracted the attention of many scholars. Based on different spatial scales, scholars have conducted in-depth analysis of the coordinated development of population urbanization and land urbanization in specific regions, and have put forward rich and scientific policy recommendations based on regional research results^6,7,30–33^. Some scholars also evaluated and analyzed the development status of population urbanization and land urbanization based on national urbanization data, and proposed strategies to address the imbalances in development^8,34–38^. In addition, Wang and Hou conducted in-depth analysis on the influence of logistics industry and local governments' behavior on the development of land-urbanization and population urbanization^39,40^. Zhu conducted empirical analysis on the mechanism of the role of population and land urbanization in economic growth and pointed out that the driving effect of population urbanization was not significant, while the impact of land urbanization on the economic development of western regions was the most significant^41^. Liu proposed that urbanization is both a process of industrial and population agglomeration, continuous economic and social development, and a process of high energy consumption and concentrated carbon emissions. The impact of China's urbanization process on total-factor carbon emission efficiency shows significant regional differences at different spatial scales^42^.

In conclusion, scholars have conducted extensive and profound research in the fields of "urbanization", "population urbanization", "land urbanization", providing rich theoretical and methodological references for this study. However, most scholars tend to explore the relationship between population urbanization and land urbanization in a specific region or at a specific scale, with few examining the coupling and coordinated development of population urbanization and land urbanization from both macro and micro perspectives and their impacts. Therefore, based on the existing research, this article takes Shaanxi Province as an example, which has a relatively moderate economic development speed and total volume nationwide. Using the entropy weight method (EWM) and the coupling coordination degree model (CCD), this study quantitatively analyzes the status and characteristics of the coupled and coordinated development of population urbanization and land urbanization in Shaanxi Province from 2010 to 2022 at both macro and micro scales. Based on the empirical results, with the assistance of the analytic network process (ANP), this study systematically identifies and evaluates the problems caused by the imbalances in the development of these two types of urbanization and proposes targeted strategies to address these issues, promoting the coordinated development of "quality" and "quantity" in new urbanization.

## Methods

### Study area overview

**Figure 1:**
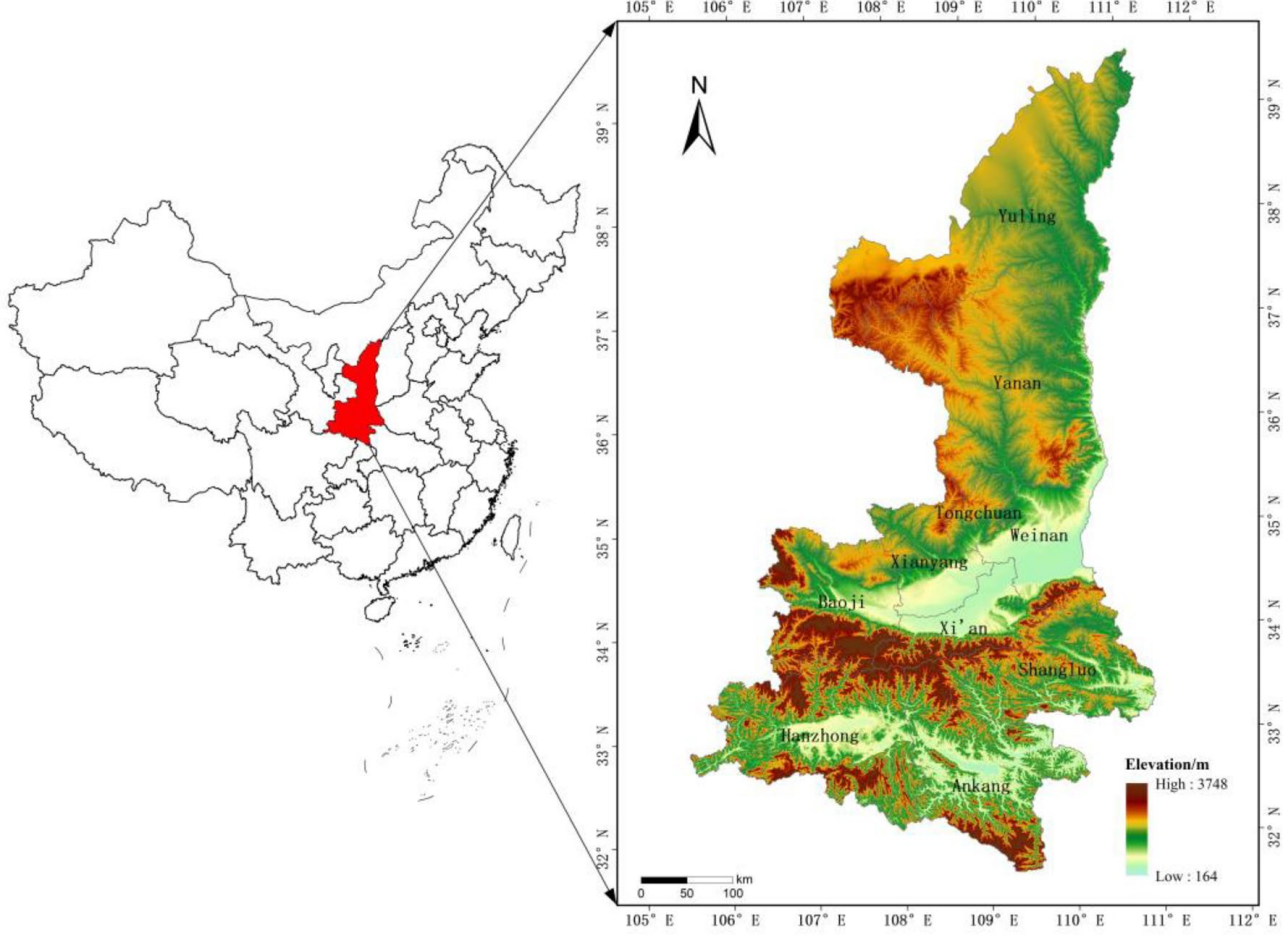
The geographical location of Shaanxi Province.

Located in the heartland of China along the middle reaches of the Yellow River, Shaanxi Province spans an area of 205,600 square kilometers. It is divided into three natural regions by the northern mountain ranges and the Qinling Mountains: the northern Shaanbei area, dominated by the Loess Plateau and comprising the cities of Yan'an and Yulin; the central Guanzhong area, characterized by the Guanzhong Plain and including the cities of Xi'an, Xianyang, Baoji, Weinan, and Tongchuan; and the southern Shaannan mountainous area, dominated by the Qinba mountains and encompassing the cities of Hanzhong, Ankang, and Shangluo. Among these, the Guanzhong region features flat terrain, high population density, and a relatively high level of socio-economic development; the Shaanbei region boasts rich energy resources such as oil, natural gas, and coal, with significant economic development potential; and the Shaannan region, rich in natural and tourism resources, lags somewhat in economic development. This mirrors the conditions of China's eastern, central, and western regions, offering a certain degree of typicality and representativeness. The geographical location of Shaanxi Province is shown in Fig. 1.

According to official data, the GDP of Shaanxi Province reached 3,272.68 billion RMB in 2022, ranking among the top in the country. By the end of 2022, the urbanization rate in Shaanxi Province reached 64.02%, narrowing the gap with the national average urbanization rate of 65.22%. In 2010, the urbanization rate in Shaanxi Province was only 45.7%. Over the past twelve years, the urbanization rate has increased by 18.32%, with an annual average urbanization growth rate of 1.36%. During the "13th Five-Year Plan" period (2016–2020), the level of urbanization in Shaanxi Province steadily increased. The urbanization rate in Shaanxi Province was 53.92% in 2015 and rose to 62.66% in 2020, an increase of 8.74%. According to the S-shaped urbanization development stage theory, the level of urbanization in Shaanxi Province is between 30 and 70%, and it is in the accelerated development stage.

### Selection of evaluation indexes and data processing

According to the definition of land urbanization and population urbanization in the article, combined with the geographical, economic, and urbanization development characteristics of Shaanxi Province, and referring to relevant literature^39,43,44^, the paper selected two variables at the target level, namely, land urbanization and population urbanization. Land urbanization was reflected in three aspects: "land structure," "land input," and "land output." Among them, "land structure" included "urban built-up area," "green coverage rate of built-up areas," and "per capita park and green space area," which respectively reflected the land use status, quality, different purposes, and distribution. "Land input" was characterized by "fixed asset investment per unit area" and "real estate investment per unit area." The limited availability of land resources made its supply relatively fixed. The investment of fixed assets within a certain area could reflect the utilization effectiveness and economic benefits of urban land. The real estate industry played an important role in the urbanization process, and its investment status could indirectly reflect the scale and benefits of human habitation in the study area. "Land output" was an assessment of the utilization effectiveness of land resources, reflecting the economic benefits and contributions of land. The article selected "value of secondary and tertiary industries per unit area" and "fiscal revenue per unit area" to characterize the land output in land urbanization. The secondary and tertiary industries were the main sectors for production and services by urban residents. By measuring the value added they created per unit area, it could reflect the economic production status of urban residents. Meanwhile, fiscal revenue per unit area reflected the level of support from decision-makers for land urbanization.

Population urbanization was characterized by four criteria-level indicators: "demographic structure," "industrial structure," "life quality of residents," and "lifestyle of residents." Among them, "demographic structure" was one of the most central indicators in previous urbanization studies, including two indicators: "proportion of non-agricultural population" and "population density." The former reflected the basic conditions of urbanization and the transition of the population from agriculture to other industries. The latter reflected the concentration of population within a unit area, reflecting the scale of urbanization. "Industrial structure" was a prominent manifestation of the concept of new urbanization and reflected the integrated development of the economy during the urbanization process. It was characterized by the "proportion of employees in the secondary and tertiary industries in urban areas" and the "proportion of value added in the secondary and tertiary industries to GDP." "Life quality of residents" and "lifestyle of residents" reflected the core concept of people-oriented in the new urbanization, taking into account the living standards and well-being of residents during the urbanization process, and they emphasized meeting the needs of residents and providing better living conditions. "Life quality of residents" was characterized by the indicators "number of higher education students per ten thousand people" and "per capita disposable income of urban residents." "Lifestyle of residents" was characterized by the indicators "number of hospital beds per ten thousand people" and "number of cars per ten thousand people."

This paper constructed a comprehensive evaluation index system for "land urbanization" and "population urbanization" in Shaanxi Province, as shown in Table 1. Considering the accessibility of data and the feasibility of statistical processing, the study selected statistical data from Shaanxi Province from 2010 to 2022. The relevant data were sourced from the "Statistical Yearbook of Shaanxi Province" and the "Statistical Yearbook of China".

**Table 1:** Comprehensive evaluation index system of new-type urbanization in Shaanxi Province.

Objective layer	Criterion layer	Index layer	Index formula and unit
Land urbanization	Land structure	Urban built-up area	square kilometers
		Green coverage rate of built-up areas	%
		Per capita park and green space area	square meters per person
	Land input	Fixed asset investment per unit area	100 million RMB yuan/square kilometer
		Real estate investment per unit area	100 million RMB yuan/square kilometer
	Land output	Value of secondary and tertiary industries per unit area	100 million RMB yuan/square kilometer
		Fiscal revenue per unit area	100 million RMB yuan/square kilometer
Population urbanization	Demographic structure	Proportion of non-agricultural population	%
		Population density	%
	Industrial structure	Proportion of employees in the secondary and tertiary industries in urban areas	%
		Proportion of value of the secondary and tertiary industries in GDP	%
	Life quality of residents	Number of higher education students per 10,000 people	RMB yuan
		Per capita disposable income of urban residents	RMB yuan
	Lifestyle of residents	Number of hospital beds per 10,000 people	–
		Number of private cars per 10,000 urban households	–

In order to exclude the influence caused by dimension and order of magnitude, this paper normalized the indicators of the plan layer, and the calculation formula was: ${Y}_{ij}=\frac{{X}_{ij}-{X}_{min}}{{X}_{max}-{X}_{min}}$. Where *Y*_*ij*_ was the normalized value, *X*_*ij*_ represented the original observed value of the *j*-th indicator under the *i*-th criterion level, *X*_*min*_ was the minimum value, and *X*_*max*_ was the maximum value.

### Evaluation of coordination level between land and population urbanization

Entropy Weight method (EWM) was an objective weighting method that could reduce subjective influences. Its principle was that the weight of each indicator was determined by the impact of its variability on the results. If an indicator had a larger range of variability, its entropy would be smaller, indicating a higher weight; conversely, if an indicator had a smaller range of variability, its entropy would be larger, indicating a lower weight. The specific calculation formula was shown as Eq. (1).

$$b_{{ij}} = Y_{{ij}} /\sum\limits_{{i = 1}}^{m} {Y_{{ij}} } ,$$

$$e_{{ij}} = - 1/{\text{ln}}\;m\sum\limits_{{i = 1}}^{m} {b_{{ij}} \;} {\text{ln}}\;b_{{ij}} ,$$

$${g}_{ij}=1-{e}_{ij},$$

1 $$w_{{ij}} = g_{{ij}} /\sum\nolimits_{{j = 1}}^{n} {g_{{ij}} }$$

In Eq. (1), ${b}_{ij}$ represented the weight of the *j*-th indicator in the *i*-th criterion layer. ${e}_{ij}$, ${g}_{ij}$ and ${w}_{ij}$ respectively represented the information entropy, coefficient of variation, and weight value of the *j*-th indicator in the *i*-th criterion layer.

Based on the comprehensive evaluation index system of land urbanization and population urbanization, a weighted average model was constructed to conduct an overall evaluation of land urbanization and population urbanization in Shaanxi Province. The standardized data ${Y}_{ij}$ and the weights ${w}_{ij}$ of each indicator were used to form a weighted average comprehensive standard model, establishing land urbanization index and population urbanization index. The comprehensive evaluation model was as follows:

2 $${L}_{L}=\sum_{i=1}^{3}\sum_{j}^{n}{w}_{ij}{Y}_{ij}$$

3 $${L}_{P}=\sum_{i=1}^{4}\sum_{j}^{n}{w}_{ij}{Y}_{ij}$$

In Eqs. (2) and (3), ${L}_{L}$ represented the land urbanization index of Shaanxi Province, reflecting the overall level of land urbanization. ${L}_{P}$ represented the population urbanization index of Shaanxi Province, reflecting the overall level of population urbanization. ${L}_{L}$ and ${L}_{P}$ were generally set to 1 when the land urbanization or population urbanization level was in an excellent state. When they were set to 0, the condition was extremely poor, and when set to 0.5, the condition was average. The larger the indicator value, the higher the level of land urbanization or population urbanization.

The Coupling Coordination Degree (CCD) model was based on the mutual interaction between two or more systems. It was used to diagnose the degree of interaction and the level of coordination between systems, and analyze the quantitative tool for the development level of coordinated development. This study drew on previous research^45^ achievements on coordination and constructed a coupling coordination evaluation model for land urbanization and population urbanization in Shaanxi Province:

4 $$C=2\times {\left[\frac{{L}_{L}\cdot {L}_{P}}{{\left({L}_{L}+{L}_{P}\right)}^{2}}\right]}^\frac{1}{2}$$

In Eq. (4): $C$ represented the coupling degree, with a value range of [0,1]. A larger value of $C$ indicated a higher degree of mutual dependence and constraint between the land urbanization and population urbanization systems.

$$T=\alpha {L}_{L}+\beta {L}_{P}$$

5 $$D=\sqrt{C\times T}$$

In Eq. (5): $T$ represented the coordination degree, with a value range of [0,1]. A larger value of $T$ indicated a better coordination status between the land urbanization and population urbanization systems. $\alpha$ and $\beta$ were the weight coefficients of the two system indices. Based on previous analysis, it was believed that land urbanization and population urbanization are equally important, so the weights were set to 0.5 each. $D$ represented the coupling coordination degree and had a value range of [0,1]. A larger value of $D$ indicated a higher level of benign coupling between the land urbanization and population urbanization systems. The coupling coordination degree was divided into ten levels, as shown in Table 2.

**Table 2:** The levels of the coupling coordination degree model.

Index layer	Index formula and unit
[0.00, 0.10)	Highly uncoordinated
[0.10, 0.20)	Moderately uncoordinated
[0.20, 0.30)	Slightly uncoordinated
[0.30, 0.40)	Basically coordinated
[0.40, 0.50)	Moderately coordinated
[0.50, 0.60)	Highly coordinated
[0.60, 0.70)	Excellent coordination
[0.70, 0.80)	Outstanding coordination
[0.80, 0.90)	Extraordinary coordination
[0.90, 1.00]	Perfect coordination

### Assessment of the impact of imbalanced development between land urbanization and population urbanization

Exploring the impact of imbalanced development between land urbanization and population urbanization, especially the negative effects and associated problems, and evaluating them is essential for coordinating their development and formulating targeted measures to alleviate imbalanced development. Adverse effects in this regard often trigger related risks, resulting in undesirable consequences presented in the form of problems. Therefore, the study **adopted** the logical method of risk identification—"field investigation and risk loss inventory"—to sort out the negative impacts of the unbalanced development of land urbanization and population urbanization in Shaanxi Province. Through field surveys, the study explored the main negative impacts and the issues that emerged, which was beneficial for acquiring firsthand data from the bottom up, ensuring the scientific rigor and comprehensiveness of problem identification. The problem list helped to clarify the approach to identifying issues caused by negative impacts in advance. The combination of these two methods was conducive to thoroughly and accurately identifying the problems caused by negative impacts.

The academic community commonly used the Fuzzy Comprehensive Evaluation (FCE) method or Analytic Network Process (ANP) to evaluate the impact of factors or to prioritize solutions to problems^46^. Although the use of the FCE was becoming more widespread, it did not necessarily yield more effective results than the simpler and more direct ANP^47^. ANP was essentially a process of structuring all the elements that influenced the outcome of a decision. By conducting pairwise numerical comparisons to assess importance and potential impact, and then integrating these results, different priorities were established^48^. ANP was able to effectively reflect the coupling relationships and feedback mechanisms between different factors based on the summation of experience and scientific prediction, making it suitable for decision analysis of complex issues. The main entities involved in the unbalanced development of land urbanization and population urbanization exhibited diverse characteristics and had widespread impacts, with certain dependencies existing among related factors. Considering the reliability and comprehensiveness of the outcomes, the study employed ANP for evaluating the impact of factors.

## Result analysis

### Result of land urbanization and population urbanization level

This paper adopted a combined method of objective and subjective evaluation to compensate for the shortcomings of single methods. The comprehensive evaluation method not only reduced the interference of subjective factors' uncertainty but also improved the accuracy and utilization rate of the original information. Both land urbanization and population urbanization were inseparable components of the "new urbanization" process, playing equally important roles in the development of "new-type urbanization." Therefore, they were both assigned a comprehensive weight value of 0.500. On this basis, the paper further calculated the comprehensive weight values of internal indicators within these two systems based on the entropy method, with the results shown in Table 3. (The raw data of each indicator and the detailed calculation process of the weights are detailed in Section 1 of the supplementary materials).

**Table 3:** The weight of each index of new urbanization in Shaanxi Province.

Objective layer	Criterion layer	Index layer	Comprehensive weight
Land urbanization (0.500)	Land structure (0.107)	Urban built-up area	0.092
		Green coverage rate of built-up areas	0.005
		Per capita park and green space area	0.010
	Land input (0.224)	Fixed asset investment per unit area	0.076
		Real estate investment per unit area	0.148
	Land output (0.169)	Value of secondary and tertiary industries per unit area	0.061
		Fiscal revenue per unit area	0.109
Population urbanization (0.500)	Demographic structure (0.085)	Proportion of non-agricultural population	0.034
		Population density	0.051
	Industrial structure (0.030)	Proportion of employees in the secondary and tertiary industries in urban areas	0.013
		Proportion of value of the secondary and tertiary industries in GDP	0.017
	Life quality of residents (0.260)	Number of higher education students per 10,000 people	0.216
		Per capita disposable income of urban residents	0.044
	Lifestyle of residents (0.124)	Number of hospital beds per 10,000 people	0.037
		Number of private cars per 10,000 urban households	0.088

From Table 3, it was evident that in the land urbanization of Shaanxi Province, the weight value of "land input" was 0.224, the highest value, the weight value of "land output" was 0.169, the second highest, and the weight value of "land structure" was 0.107, the lowest. This meant that the effective utilization and optimal allocation of land resources played a crucial role in promoting the urbanization development of the province. Governments should focus on land management, improving land use efficiency through rational planning and utilization of land resources, thereby promoting economic development and increasing fiscal revenue. Secondly, land output held a higher proportion in the weight values. This indicated that in the urbanization process of the province, the use effect and economic benefits of land were equally important. By improving the investment in fixed assets per unit area and real estate investment per unit area, land output could be increased and economic growth could be promoted. Therefore, it was necessary to strengthen the planning and management of land development and the real estate industry to ensure the full use and potential of land resources. Lastly, while land structure held a lower position in the weight values, it still had certain importance. Land structure involved the diversity and rationality of land use; by optimizing the land structure, the city's functionality and competitiveness could be enhanced, promoting the transformation and upgrading of the economic structure.

In population urbanization in Shaanxi Province, the weight value of "life quality of residents" was 0.260, which was the highest; the weight value of "lifestyle of residents" was 0.124, which was the second-highest; the weight value of "demographic structure" was 0.085, relatively lower; and the weight value of "industrial structure" was 0.030, which was the lowest. It could be seen that improving the quality of life for residents was the most important goal in the process of population urbanization in this region. This meant that governments should focus on and improve the material and non-material living conditions of residents, including providing high-quality education, creating employment opportunities, and improving housing conditions. The significant weight value of lifestyle indicated that shaping a positive, healthy, and sustainable lifestyle was also one of the important goals in population urbanization. Although population structure held a relatively lower position in the weight values, it still carried certain significance. By planning and adjusting the population structure reasonably, diverse needs of different population groups could be better met, promoting sustainable development of urbanization, economic development, and social progress. Industrial structure held a relatively lower position in the weight values, which suggested that although the adjustment and transformation of the industrial structure were important in the process of population urbanization in the region, its role was relatively less prominent. However, this did not undermine the positive effects of reasonable adjustment of the industrial structure in promoting economic growth and increasing employment opportunities.

**Figure 2:**
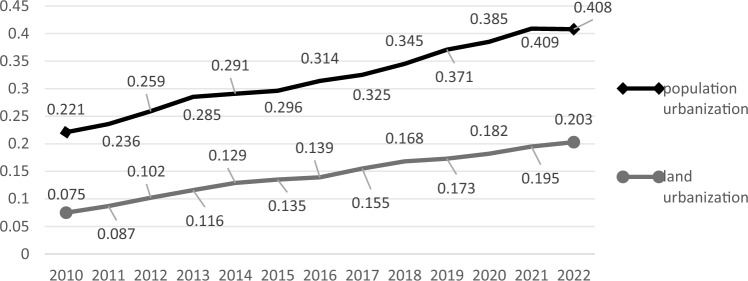
Level of land urbanization index and population urbanization in Shaanxi Province from 2010 to 2022.

Based on the weight values of land urbanization and population urbanization indicators in Shaanxi Province, this study, through the calculation of the weighted average model, further conducted a comprehensive evaluation and analysis of the level of land urbanization and population urbanization in the province. The calculation results were shown in Fig. 2.

Between 2010 and 2022, the level of land urbanization in Shaanxi Province was significantly lower than the level of population urbanization, but both showed a steady upward trend. The land urbanization index ranged from 0.075 to 0.203, while the population urbanization index was between 0.221 and 0.408. Moreover, the increase in the land urbanization index was only 0.128, whereas the increase in the population urbanization index reached 0.187. This phenomenon indicates that although Shaanxi Province made rapid progress in population urbanization, the development of land urbanization still faced significant challenges and was lagging. Possible reasons include the relatively limited supply of land resources, low efficiency in land use, and inadequate adjustment of land structure.

### Results of the coupling and coordinated development of land urbanization and population urbanization

**Figure 3:**
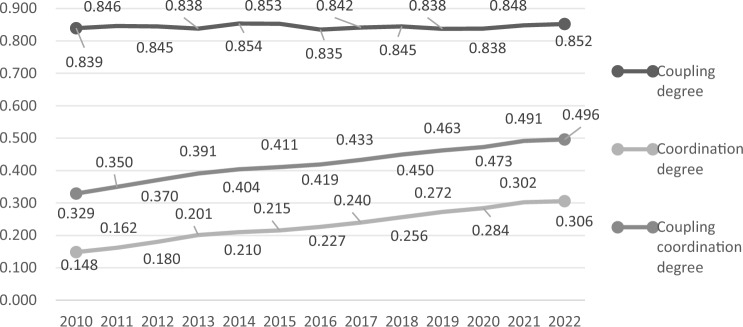
The coupling degree, coordination degree, and coupling coordination degree of land urbanization and population urbanization in Shaanxi Province from 2010 to 2022.

From the measurement results of the levels of land urbanization and population urbanization in the study area from 2010 to 2022, it was found that the overall levels of land urbanization and population urbanization in Shaanxi Province were relatively low. To explore the specific conditions of the coupled and coordinated development of land urbanization and population urbanization in the province, the study measured the coupling degree, coordination degree, and the degree of coupling coordination between them. The measurement results were shown in Fig. 3. (The calculation results of the coupling coordination degree between land urbanization and population urbanization of each city in the study area were detailed in Section 2 of the supplementary materials).

Between 2010 and 2022, the level of coupling between land urbanization and population urbanization in Shaanxi Province was relatively high, and the range of change was small. The degree of coordination was lower, showing a slowly increasing trend. The degree of coupling and coordination was relatively low, also constrained by the degree of coordination, but it was on an upward trend. This indicates that there was an imbalance in the development between land urbanization and population urbanization in the province. Although there was a high degree of interdependence between the two, the overall consistency of development was poor.

**Figure 4:**
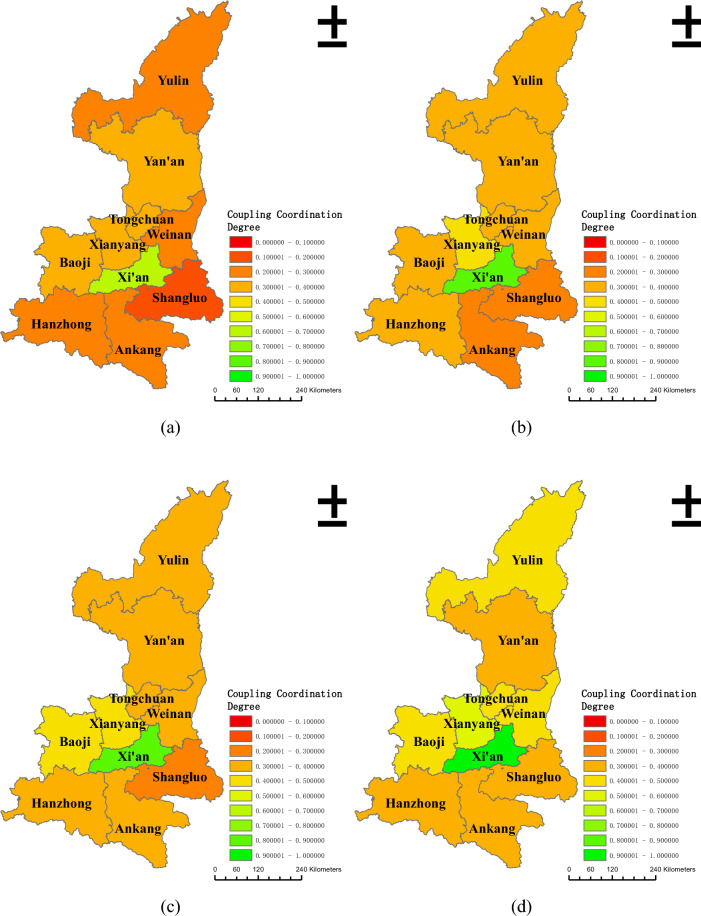

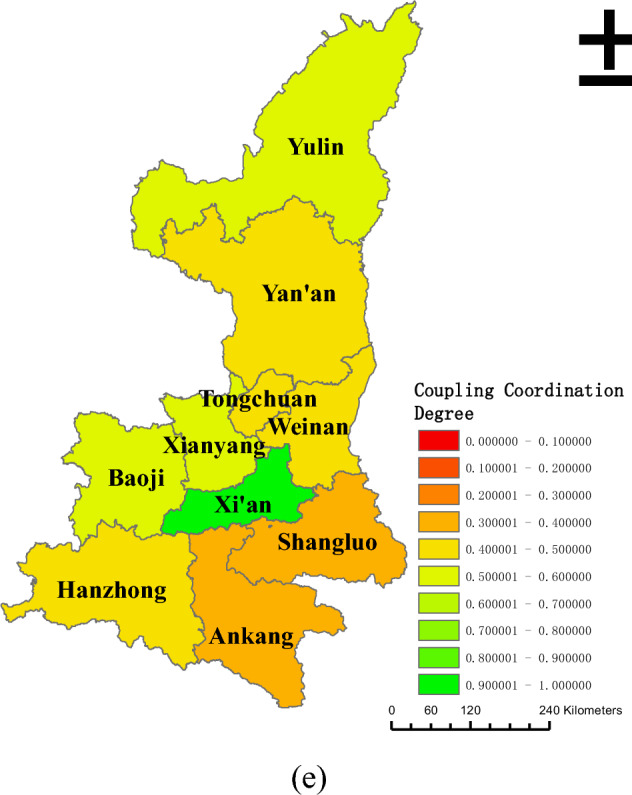
Coupling and coordination degree of land urbanization and population urbanization in Shaanxi Province from 2010 to 2022. (**a**–**e**) correspond to the years 2010, 2013, 2016, 2019, and 2022.

In order to further explore the coordinated development of land urbanization and population urbanization in the province, ArcGis 10.8 was used in the study to correlate the coupling coordination degree of different cities in Shaanxi Province with the administrative division map of Shaanxi Province. Based on the classification of different coupling coordination levels in Table 2, spatial distribution maps of the coupling coordination degree of land urbanization and population urbanization in Shaanxi Province in 2010, 2013, 2016, 2019, and 2022 were created, as shown in Fig. 4.

Between 2010 and 2022, the spatial distribution of the coupling and coordination degree of land urbanization and population urbanization in Shaanxi Province generally showed characteristics of being "high in the middle and low in the north and south." The level of coupled and coordinated development in the Guanzhong area was high, followed by the northern Shaanxi region, with the southern Shaanxi region being the lowest. In 2010, only Xi'an reached a primary coordination level, except for Shangluo, which was in a state of severe imbalance, with the rest of the cities being in a state of mild or severe imbalance. By 2013, Xi'an achieved and maintained a good coordination state, with only Xianyang being on the verge of imbalance, while the rest of the cities remained in mild or moderate imbalance. From 2013 to 2019, more than half of the prefecture-level cities in Shaanxi Province reached or exceeded a state of being on the verge of imbalance. By the end of 2019, only Yan'an, Hanzhong, Ankang, and Shangluo were in a state of mild imbalance, while Xi'an reached a state of excellent coordination. In 2022, Baoji, Xianyang, and Yulin all achieved a barely coordinated state, with only Ankang and Shangluo still in a state of mild imbalance. Except for Xi'an, which continued to maintain an excellent coordination state, the rest of the cities reached a state of being on the verge of imbalance.

On a macro scale, the degree of interdependence between land urbanization and population urbanization in Shaanxi Province was significant, with the coupling degree consistently maintaining above 0.835 throughout the sample period, but the degree of benign coupling was low, with the coordination degree remaining below 0.306 during the sample period. On a micro scale, the "high in the middle and low in the north and south" characteristic was displayed by the coupling and coordination degree. This indicates that the development of the two types of urbanization in Shaanxi Province lacked interactive consistency and effectiveness, and there were still issues of uneven development within the region. Different areas possess different resource endowments and advantages. Through regional coordinated development, the optimization of resource allocation can be achieved, and by rationally planning and utilizing the industries, manpower, capital, and natural resources of each area, not only can the level of balanced development of regional urbanization be improved, but it can also guide the transfer of population and industrial resources to relatively weaker areas, alleviate the pressure on large cities, and achieve balanced development of urban agglomerations.

Additionally, as the central city of Shaanxi Province, Xi'an exhibited a high degree of coupling and coordination between land urbanization and population urbanization, creating a strong attraction and developmental advantages. This led to the population and resources from surrounding cities flowing more towards Xi'an, resulting in a siphon effect of population and resources. It exacerbated the imbalance in development between regions. This situation was not unique to Shaanxi but was present in many provinces across China, showcasing its representativeness. Addressing the issue of central cities excessively attracting population and resources could promote the balanced development of regional urbanization by reasonably dispersing population and resources across various cities. It could also leverage the unique advantages of different cities and the radiating role of central cities. Therefore, the problems of regional development imbalance and the weak radiating effect of central cities needed to be given attention.

### The problems caused by the imbalanced development of land urbanization and population urbanization, and their assessment

Between 2010 and 2022, the development of land urbanization in Shaanxi Province lagged behind that of population urbanization. This study, with the aid of field inspections and the Risk and Loss Inventory method, identified the issues arising from the unbalanced development of the two, which led to negative impacts.

Firstly, urban expansion space was limited. Due to the relatively limited availability of land resources for development, the rapid development of population urbanization led to an increase in the demand for residential and working spaces. This made it difficult for existing land resources to meet the needs of rapidly growing populations, leading to relative overcrowding and housing shortages, thereby increasing the difficulty of further urban expansion. Additionally, land use planning and management mechanisms were not robust, resulting in low efficiency of land development and utilization. In some areas, there were instances of land lying idle and farmland being illegally occupied, further increasing the difficulty of urban expansion.

Secondly, urban infrastructure support lagged behind. The delay in land urbanization meant that cities faced greater difficulties in providing residents with sufficient roads, bridges, water, electricity, and other basic infrastructure, leading to an inadequate supply of infrastructure. Moreover, the delay in land urbanization, which resulted in insufficient utilization of land resources, reduced the economic benefits of land development. This, in turn, decreased the sources of funding for governments to construct urban infrastructure. Additionally, the delay in land urbanization also highlighted the uneven distribution of urban infrastructure; while infrastructure construction in central urban areas was adequate but limited in space, the infrastructure in suburbs and areas far from the city center was insufficient.

Thirdly, it exacerbated the degree of social inequity. The delay in land urbanization led to the uneven distribution of urban resources and an imbalance between the supply and demand for land. This resulted in the concentration of the value and usage rights of land in the hands of a few, who then had access to more land resources and opportunities for wealth, intensifying the disparity between the wealthy and the poor and contributing to social injustice. Moreover, the overly rapid development of population urbanization could further strain urban housing supply. Under market conditions, this made it more challenging for low-income families or vulnerable groups to secure suitable housing, potentially worsening the issue of social housing inequity and even becoming one of the contributors to social instability.

Fourthly, the ecological environment was damaged. The delay in land urbanization made urban expansion more challenging. In the short term, cities would extend into the surrounding rural areas, leading to the development and transformation of previously intact natural ecosystems such as farmland, forests, and wetlands. Such land use changes could destroy the original ecosystems, leading to the loss of biodiversity and ecological functions. Furthermore, the delay in land urbanization could result in fragmented regional construction activities, damaging the original ecological patterns and disrupting ecological balance. The delay in land urbanization also damaged, to some extent, urban ecological services such as water conservation, air purification, and climate regulation, adversely affecting the quality of life and health of urban residents.

**Figure 5:**
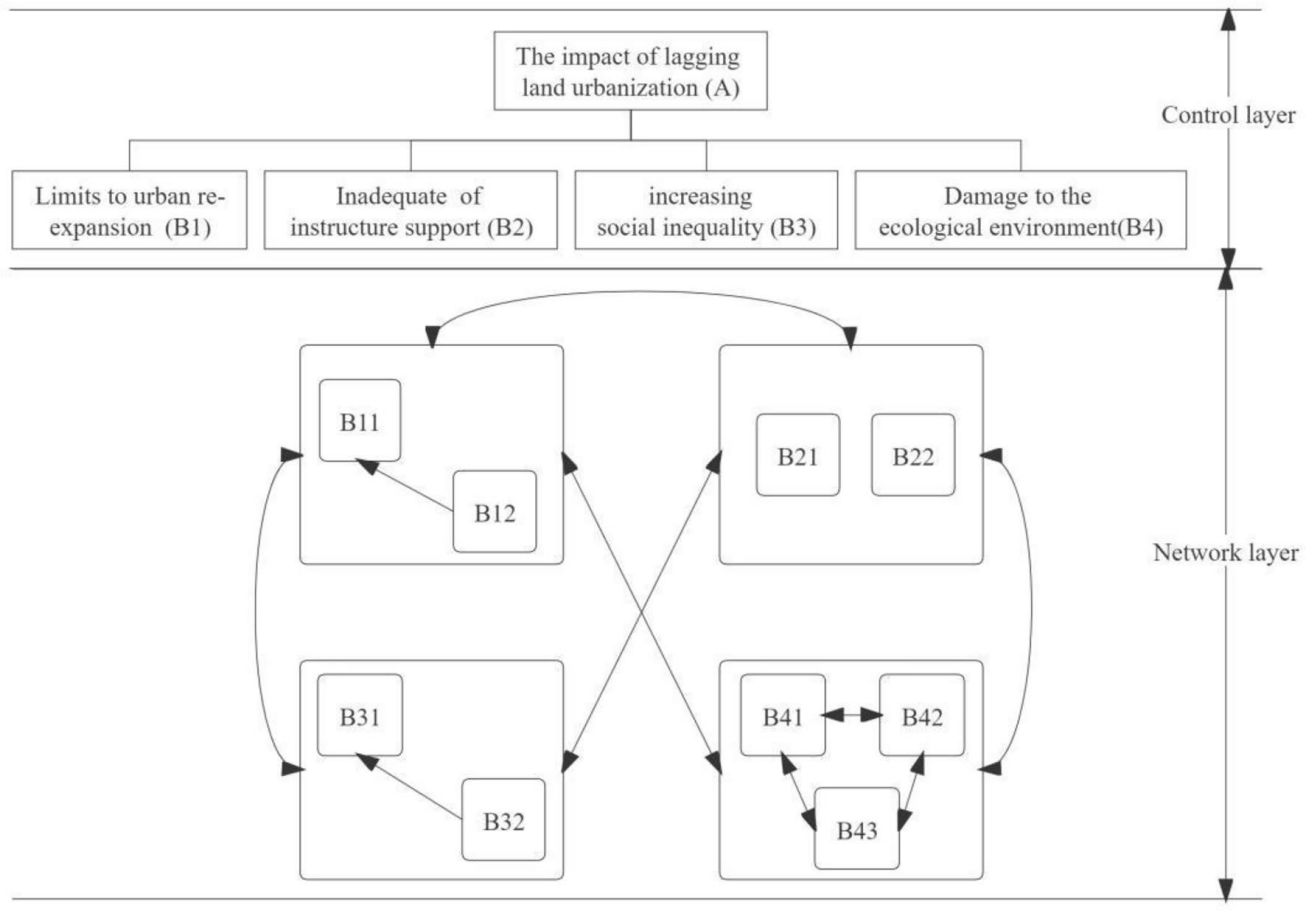
Evaluation model of the impacts or problems caused by the lagging land urbanization in Shaanxi Province.

Based on the analysis provided, an ANP model's indicator system could be constructed, as shown in Table 4. The foundation of constructing a Network Hierarchical Model was the relationships of mutual influence between elements within a set, between elements across different sets, and the feedback or dependency connections among evaluation indicators. Considering the interrelations among the various elements related to the lag in urbanization of land in Shaanxi Province, an assessment model for the issues caused by the urbanization lag in Shaanxi Province was constructed, as illustrated in Fig. 5.

**Table 4:** The indicator system for the problems caused by the lagging land urbanization in Shaanxi Province.

Objective layer	Criterion layer	Network layer
Effects or problems caused by the lag of land urbanization (A)	Limits to urban re-expansion (B_1_)	Scarcity of land resources (B_11_)
		Irrationality of land planning (B_12_)
	Inadequate of infrastructure support (B_2_)	Undersupply of infrastructure (B_21_)
		Maldistribution of infrastructure (B_22_)
	Increasing social inequality (B_3_)	Widening gap between rich and poor (B_31_)
		Social housing inequality (B_32_)
	Damage to the ecological environment (B_4_)	Destruction of ecosystem (B_41_)
		Disturbance of ecological balance (B_42_)
		Weakening of ecological service function (B_43_)

Next, we constructed judgment matrices, applying a 1–9 scale method to measure the dominance among elements, resulting in the determination of element weights and passing the consistency test results. On this basis, using the Super Decision (v3.2) software, the calculation obtained the limit supermatrix of factors causing issues due to the lag in urbanization of land in Shaanxi Province. By synthesizing the elements related to the problems caused by the lag in urbanization of land, the final ranking of the weights of each factor was obtained, which is the limit supermatrix of factors causing issues in urbanization of land in Shaanxi Province, as shown in Table 5. (The calculation process of the ANP model was detailed in Section 3 of the supplementary material).

**Table 5:** The extreme supermatrix of the factors of the problems caused by the lagging land urbanization in Shaanxi Province.

	B_11_	B_12_	B_21_	B_22_	B_31_	B_32_	B_41_	B_42_	B_43_
B_11_	0.121	0.121	0.121	0.121	0.121	0.121	0.121	0.121	0.121
B_12_	0.275	0.275	0.275	0.275	0.275	0.275	0.275	0.275	0.275
B_21_	0.174	0.174	0.174	0.174	0.174	0.174	0.174	0.174	0.174
B_22_	0.098	0.098	0.098	0.098	0.098	0.098	0.098	0.098	0.098
B_31_	0.006	0.006	0.006	0.006	0.006	0.006	0.006	0.006	0.006
B_32_	0.154	0.154	0.154	0.154	0.154	0.154	0.154	0.154	0.154
B_41_	0.022	0.022	0.022	0.022	0.022	0.022	0.022	0.022	0.022
B_42_	0.009	0.009	0.009	0.009	0.009	0.009	0.009	0.009	0.009
B_43_	0.141	0.141	0.141	0.141	0.141	0.141	0.141	0.141	0.141

Through the aforementioned steps, the weight values of the indicators for factors causing issues due to the lag in urbanization of land in Shaanxi Province were obtained, namely the ranking of the priority levels of various problems to be addressed. From the perspective of the criteria layer indicators, the issue of "limits to urban re-expansion" had a weight value of 0.396, ranking it at the forefront, indicating it was a problem that required significant attention and resolution. This issue was specifically manifested in irrational land planning and the scarcity of land resources. The issue of "irrationality of land planning" had a composite weight value of 0.275, ranking first among the nine network layer indicators. Rational land planning not only met the demands of urban development for different types of land use and avoided disorderly urban sprawl and land waste. This indicator, being the highest-ranked, indicated that land planning was a core measure to promote orderly development of land urbanization and was the most pressing issue to be addressed in Shaanxi Province at the time. The issue of "scarcity of land resources" ranked fourth from the bottom in the network layer, which was related to the characteristics of the issue itself. Population and economic activities concentrated in cities during the urbanization process, continuously increasing the demand for land resources and exacerbating land scarcity. Moreover, competition for land among different uses became more intense, further intensifying land resource scarcity. Alleviating land scarcity, improving land use efficiency, and appropriately increasing land use intensity were effective approaches to drive urban regeneration and sustainable development of land urbanization. This issue was prevalent in many provinces in China.

The issue of "inadequate of infrastructure support" was the second most pressing to be addressed, with a weight value of 0.272. The most prominent issue was the "Undersupply of infrastructure", which had a weight value of 0.174, while the issue of "maldistribution of infrastructure" was relatively less severe, with a weight value of 0.098. As the "skeleton" of the city, an adequate supply and efficient construction of infrastructure could effectively alleviate problems such as traffic congestion, insufficient public services, water scarcity, and tense energy supplies during the urbanization process. Moreover, an equitable distribution of infrastructure was a powerful means to promote balanced development of new urbanization in regions, improve regional competitiveness, and enhance the quality of life and social equity for urban residents. Therefore, alleviating the issue of limited urban expansion and improving the insufficient infrastructure support were urgent tasks in the urbanization process at that time.

The issue of "damage to the ecological environment" was relatively less urgent to address, with a weight value of 0.172. As other problems were resolved, this issue was somewhat alleviated. The most significant problem was the "weakening of ecological service function", which had a weight of 0.141 and ranked fourth. Ecological service functions are crucial for improving urban environmental quality, enhancing natural disaster defense capabilities, improving residents' quality of life, and promoting social interaction and community cohesion, all essential to land urbanization. Due to the relatively simple balance of urban ecosystems, the severity of "destruction of ecosystem" and "disturbance of ecological balance" issues was relatively lower but still not to be overlooked. Ensuring the health and balance of ecosystems played a significant role in enhancing residents' quality of life, mitigating natural disasters, and securing natural resources for urbanization.

Issues of "increasing social inequality" often stemmed from the negative impacts of other related parties, hence the severity of this problem was relatively lower, with a weight value of 0.160. The resolution of other issues might weaken the impact of this problem. However, "social housing inequality" stood out among the nine issues, with a weight value of 0.154. In the Chinese mindset, housing often signified "family". It was not only an important form of asset and a long-term investment channel but also considered a symbol of social status. The fairness of social housing not only could reduce the housing difficulties faced by low-income or disadvantaged groups, alleviating social conflicts and inequality issues, but also could reasonably guide population movement, avoid excessive concentration of population, relieve urban pressure, and promote the rational development of land urbanization. Therefore, curbing the "damage to the ecological environment" and the "increasing social inequality" were also important tasks at the time.

## Discussion

This study, based on provincial panel data, selected provinces in China that were relatively moderate in terms of economic development speed and total volume and had significant geographical and demographic characteristics as evidence objects. It evaluated the coordinated development status of population urbanization and land urbanization in Shaanxi Province from both macro and micro perspectives. It was found that Shaanxi Province had made rapid progress in population urbanization, whereas the development of land urbanization faced significant challenges and lagged behind. The interdependence between the two was strong, but their development consistency was poor. The spatial distribution of the coupling and coordination degree of population urbanization and land urbanization within the province generally exhibited a characteristic of "high in the middle, low in the south and north". This study's consideration of the urbanization development status in the research area could, to a certain extent, enrich the local research system and obtain more general patterns and conclusions, possessing a certain reference value. It could provide reference value for solving such problems in the urbanization process of most developing countries.

Furthermore, this study did not merely stay at the level of evaluating the coordinated development status between population urbanization and land urbanization. On this basis, it further identified the issues caused by the lag of land urbanization behind population urbanization through the evaluation results. Among these, the issues of restricted urban expansion and insufficient infrastructure support were urgent to address, the problems of ecological environment damage and exacerbated social injustice could not be overlooked, and the issues of regional development imbalance and the weak radiating effect of central cities needed attention. This was a crucial step from theoretical analysis to decision-making implementation.

However, despite the achievements mentioned above, this study had some shortcomings. First, there were sample limitations. Due to the relatively short span of available samples, the conclusions of this study might have certain limitations. Future research could enhance the generalizability of the study by expanding the sample size and using diversified samples. Second, the ANP method used in this study to identify and evaluate the problems caused by the lag of land urbanization behind population urbanization in Shaanxi Province, although capable of achieving the study's objective of comparing and ranking these issues, had inherent subjectivity due to the ANP's procedural nature. Future studies might consider using gray ANP or fuzzy ANP methods to overcome this limitation, in order to obtain more accurate and comprehensive results.

## Conclusion

Based on the empirical analysis of population urbanization and land urbanization from 2010 to 2022, the study believed that to advance the construction of new urbanization in Shaanxi Province, further improve the coordination between population urbanization and land urbanization, and reduce the negative impact brought about by the developmental imbalance between the two, measures should have been taken from the following aspects.

### Optimize land spatial layout and strengthen infrastructure support

To alleviate the issue of restricted urban expansion caused by the lag in land urbanization development in Shaanxi Province and achieve layout optimization within a limited urban area, relying solely on the limited rational decisions of relevant departments is insufficient. It is also necessary to combine modern scientific and technological means for predictive analysis. Photogrammetry and remote sensing technologies should be leveraged to observe data on land resources, training reliable AI models to comprehensively predict the direction of future urban expansion. By integrating big data to capture consumer preferences, it facilitates rational spatial layouts for industries and residences. Moreover, through the renovation of old urban areas, redevelopment of underutilized lands, and transformation of industrial sites, the utilization efficiency and economic output of existing urban lands can be enhanced. In the rural land market, implementing a price disclosure system and a collective decision-making system stimulates land market vitality, promotes the circulation of rural land, and encourages collective land management by farmers.

To address the insufficient support for urbanization infrastructure in Shaanxi Province, governments should, based on an appropriate increase in fiscal budget, gradually expand investment in a timely manner, such as charging for the use of certain infrastructure, leasing the overall development and operating rights of regions within a specified period to attract social capital participation and seek related international cooperation, thereby diversifying the sources of infrastructure construction funds. Additionally, infrastructure construction should be strengthened from a systemic level by implementing a responsibility system, integrating annual urban construction into a work mechanism that binds engineering quality progress and target responsibilities, with relevant responsible persons signing quality indicators and engineering progress target responsibility documents.

### Enhance the urban ecological service function and improve residents' quality of life

The delayed urban land development in Shaanxi Province has also led to improvements in ecological and environmental issues, which are closely related to the enhancement of urban ecosystem service functions. It necessitates that governments establish comprehensive urban green space protection policies and regulations, clarifying the protection scope, usage restrictions, and protection measures of urban green spaces, and strengthening the supervision and enforcement of green spaces. Additionally, water resource management should be enhanced by formulating reasonable water supply plans, protecting and conserving water resources, and promoting the diversified use of water resources, such as rainwater collection and utilization, and the recycling of water resources. By strengthening environmental regulation, encouraging enterprises to implement energy-saving and emission-reduction measures, adopting clean production technologies, reducing the emissions of waste gas and wastewater, promoting public transportation and non-motorized travel, and reducing automobile exhaust emissions and noise pollution to the ecosystem, these ecological issues can be effectively improved.

The issues of social injustice exacerbated by the lagging urban land development in Shaanxi Province are mostly derived from other aspects. Therefore, on top of addressing the spatial layout of land, infrastructure, and urban ecosystem issues, it is crucial to focus on improving the quality of life for urban residents to fundamentally solve the problem of increasing social injustice. For example, governments, based on demand and population distribution, should allocate infrastructure resources rationally to ensure an equitable distribution of resources among various regions and communities. It should provide high-quality educational resources, including universal compulsory education, good school facilities, and teaching conditions, to ensure that children from different communities in the city have equal educational opportunities. Furthermore, it should formulate reasonable housing policies to secure the basic housing rights of low-income populations, offering affordable housing options.

### Provide multidimensional support to low-coordination regions and strengthen the radiation effect of central cities

The spatial distribution of the coupling coordination degree between land urbanization and population urbanization in Shaanxi Province reflected the characteristic of uneven regional urbanization development. In response, support for low-coordination areas should be strengthened by formulating targeted preferential policies based on the actual conditions of these areas, such as increasing the level of transfer payments, offering certain tax incentives, and giving priority to supporting investment and construction projects. The locational and resource advantages should be fully leveraged, creating industry chains that match local characteristics to promote the cultivation and development of emerging industries and increase employment opportunities. It aims to facilitate the synergistic development of coordinated and non-coordinated regions through promoting the cross-regional flow of resources, industries, and labor, achieving complementary advantages, and forming a regional linkage effect. This promotes intensive utilization of resources and collaborative development between regions, pushing for the coordinated progress of land urbanization and population urbanization. At the same time, central cities and surrounding cities should develop overall development plans and specialized plans in related fields, such as urban planning, transportation planning, and social affairs planning, clarifying each city's positioning and functional division, forming regional synergy, and enhancing the comprehensive competitiveness of the entire region.

In many developing countries around the world, urban expansion difficulties, inadequate infrastructure support, ecological environment damage, and exacerbation of social injustices are common issues to a certain extent due to land urbanization lagging behind population urbanization. The core solutions to these problems involve improving the efficiency of land resource use, ensuring infrastructure meets the demands of urbanization, guaranteeing the integrity of urban ecological service functions, and enhancing the quality of life for urban residents.

### Supplementary Information

Supplementary Information.

## Data Availability

The data are available from the corresponding author on reasonable request. [Name: Ai Xinnan, E-mail: 2021103030202@stu.nwupl.edu.cn].
