# Supplementary material for: Research on the coordinated development between land urbanization and population urbanization in Shaanxi Province, China
Source: Sci Rep. 2024 Apr 4;14:7977. doi: 10.1038/s41598-024-58625-3 (PMC10995124; doi:10.1038/s41598-024-58625-3)
Supplement: Supplementary file 1 — Supplementary Information. [file 41598_2024_58625_MOESM1_ESM.docx]

**Raw data of indicators and related calculation process**

**1. Original data of the indicators and the calculation process of the entropy weight method**

**1.1 Original data for indicators of population urbanization and land urbanization in Shaanxi Province from 2010 to 2022.**

To facilitate layout arrangement, city names in the original data table are represented by uppercase letters, with their meanings as follows: **A** represents Xi'an, **B** represents Tongchuan, **C** represents Baoji, **D** represents Xianyang, **E** represents Weinan, **F** represents Yan'an, **G** represents Hanzhong, **H** represents Yulin, **I** represents Ankang, and **J** represents Shangluo. The names of the indicators are represented by lowercase letters, with their meanings as follows: **a** represents Urban built-up area, **b** represents Green coverage rate of built-up areas, **c** represents Per capita park and green space area, **d** represents Fixed asset investment per unit area, **e** represents Real estate investment per unit area, **f** represents Value of secondary and tertiary industries per unit area, **g** represents Fiscal revenue per unit area, **h** represents Proportion of non-agricultural population, **i** represents Population density, **j** represents Proportion of employees in the secondary and tertiary industries in urban areas, **k** represents Proportion of value of the secondary and tertiary industries in GDP, **l** represents Number of higher education students per 10,000 people, **m** represents Per capita disposable income of urban residents, **n** represents Number of hospital beds per 10,000 people, **o** represents Number of private cars per 10,000 urban households.

Tab.1 Original data for indicators of population urbanization and land urbanization in Shaanxi Province from 2010 to 2022

| **City/Year** | **a** | **b** | **c** | **d** | **e** | **f** | **g** | **h** | **i** | **j** | **k** | **l** | **m** | **n** | **o** |
| --- | --- | --- | --- | --- | --- | --- | --- | --- | --- | --- | --- | --- | --- | --- | --- |
| **2022** |  |  |  |  |  |  |  |  |  |  |  |  |  |  |  |
| **A** | 807.570 | 43.940 | 12.020 | 0.901 | 2557.761 | 11162.930 | 0.083 | 79.590 | 8064.520 | 99.890 | 97.183 | 642.972 | 48418.000 | 62.424 | 3020.337 |
| **B** | 48.850 | 40.240 | 12.010 | 0.184 | 122.710 | 469.640 | 0.006 | 64.410 | 7890.910 | 99.800 | 92.897 | 79.261 | 38327.000 | 100.312 | 1451.376 |
| **C** | 117.590 | 43.550 | 15.010 | 0.292 | 163.499 | 2508.730 | 0.005 | 59.420 | 5926.660 | 99.850 | 91.456 | 120.421 | 40388.000 | 91.877 | 1374.797 |
| **D** | 78.150 | 39.640 | 17.480 | 0.324 | 370.195 | 2409.500 | 0.011 | 57.610 | 7391.300 | 99.990 | 85.518 | 385.719 | 42542.000 | 78.067 | 1092.821 |
| **E** | 68.360 | 41.390 | 15.340 | 0.184 | 203.087 | 1770.400 | 0.007 | 51.340 | 2073.410 | 99.100 | 80.431 | 43.225 | 39529.000 | 75.690 | 1381.085 |
| **F** | 70.970 | 41.290 | 12.670 | 0.027 | 23.160 | 2005.440 | 0.005 | 61.970 | 2360.710 | 99.940 | 89.853 | 125.027 | 40938.000 | 68.866 | 1870.127 |
| **G** | 66.880 | 41.420 | 15.540 | 0.081 | 69.209 | 1613.150 | 0.002 | 52.780 | 4336.830 | 99.570 | 84.660 | 141.509 | 38776.000 | 88.632 | 1282.553 |
| **H** | 78.380 | 38.810 | 16.300 | 0.045 | 38.032 | 6231.400 | 0.022 | 62.300 | 3716.200 | 99.870 | 95.228 | 94.467 | 40355.000 | 62.855 | 2367.952 |
| **I** | 45.000 | 42.050 | 12.100 | 0.067 | 66.822 | 1094.090 | 0.001 | 52.270 | 2219.380 | 99.930 | 86.240 | 114.914 | 31976.000 | 78.454 | 981.966 |
| **J** | 26.000 | 44.860 | 14.990 | 0.088 | 23.521 | 776.800 | 0.001 | 49.980 | 6232.500 | 99.790 | 86.066 | 106.183 | 29931.000 | 79.932 | 606.864 |
| **2021** |  |  |  |  |  |  |  |  |  |  |  |  |  |  |  |
| **A** | 805.600 | 43.000 | 11.850 | 0.816 | 2402.685 | 10379.460 | 0.085 | 79.490 | 7855.450 | 99.890 | 97.760 | 761.749 | 46931.000 | 59.852 | 2867.045 |
| **B** | 48.850 | 40.000 | 12.480 | 0.167 | 119.735 | 423.490 | 0.007 | 64.200 | 7470.910 | 99.800 | 93.450 | 78.013 | 36588.000 | 102.908 | 1369.376 |
| **C** | 113.750 | 43.370 | 14.220 | 0.267 | 212.024 | 2371.030 | 0.005 | 58.740 | 5938.470 | 99.850 | 95.440 | 117.900 | 38741.000 | 89.793 | 1280.055 |
| **D** | 76.220 | 39.520 | 15.890 | 0.292 | 390.463 | 2221.820 | 0.011 | 57.250 | 7942.030 | 99.990 | 98.270 | 375.680 | 40846.000 | 76.520 | 1049.869 |
| **E** | 68.310 | 41.100 | 14.920 | 0.200 | 290.175 | 1695.430 | 0.007 | 51.070 | 2081.650 | 99.100 | 87.680 | 42.434 | 37772.000 | 73.705 | 1310.838 |
| **F** | 70.400 | 39.240 | 12.070 | 0.028 | 33.501 | 1818.430 | 0.004 | 61.800 | 2268.630 | 99.940 | 92.520 | 122.966 | 39306.000 | 67.699 | 1754.184 |
| **G** | 60.420 | 39.770 | 15.520 | 0.072 | 79.812 | 1486.600 | 0.002 | 52.260 | 4252.510 | 99.570 | 90.860 | 158.841 | 37123.000 | 85.345 | 1182.375 |
| **H** | 78.380 | 38.120 | 16.240 | 0.042 | 38.127 | 5315.430 | 0.014 | 62.100 | 3618.440 | 99.870 | 96.240 | 93.200 | 38451.000 | 62.102 | 2197.239 |
| **I** | 45.000 | 40.280 | 12.100 | 0.065 | 87.306 | 1048.880 | 0.001 | 51.600 | 2218.750 | 99.930 | 89.920 | 113.230 | 30496.000 | 77.715 | 907.166 |
| **J** | 26.000 | 44.610 | 15.080 | 0.074 | 29.097 | 727.910 | 0.001 | 49.420 | 6087.500 | 99.790 | 86.850 | 104.603 | 28655.000 | 79.218 | 567.999 |
| **2020** |  |  |  |  |  |  |  |  |  |  |  |  |  |  |  |
| **A** | 700.690 | 41.850 | 11.850 | 0.923 | 2596.434 | 9710.980 | 0.072 | 79.200 | 7047.100 | 99.890 | 97.530 | 718.596 | 43713.000 | 57.876 | 2598.946 |
| **B** | 48.850 | 39.910 | 12.100 | 0.155 | 87.237 | 345.370 | 0.006 | 63.670 | 7496.360 | 99.800 | 93.060 | 75.354 | 34143.000 | 96.338 | 1295.676 |
| **C** | 97.940 | 41.050 | 12.500 | 0.258 | 189.035 | 2076.570 | 0.005 | 57.040 | 6063.980 | 99.850 | 96.700 | 113.311 | 36209.000 | 86.327 | 1164.559 |
| **D** | 74.680 | 39.800 | 16.030 | 0.265 | 254.077 | 1836.820 | 0.009 | 55.440 | 7818.840 | 99.990 | 98.740 | 388.815 | 37975.000 | 77.504 | 1094.401 |
| **E** | 68.290 | 40.470 | 14.920 | 0.218 | 304.852 | 1482.730 | 0.006 | 49.310 | 2056.180 | 99.100 | 86.650 | 40.770 | 35304.000 | 71.278 | 1228.040 |
| **F** | 41.000 | 40.770 | 12.540 | 0.037 | 42.388 | 1402.210 | 0.004 | 61.370 | 1949.170 | 99.940 | 94.960 | 118.920 | 36577.000 | 66.626 | 1633.804 |
| **G** | 57.600 | 39.030 | 14.750 | 0.060 | 69.789 | 1331.730 | 0.002 | 50.960 | 4148.440 | 99.570 | 90.510 | 153.446 | 34417.000 | 79.863 | 1065.437 |
| **H** | 78.380 | 37.880 | 16.630 | 0.049 | 32.009 | 3871.450 | 0.009 | 61.600 | 3529.050 | 99.870 | 95.400 | 74.645 | 35682.000 | 60.364 | 2013.058 |
| **I** | 45.000 | 40.150 | 12.070 | 0.061 | 74.504 | 932.910 | 0.001 | 49.920 | 2216.250 | 99.930 | 89.360 | 94.273 | 28247.000 | 70.819 | 821.481 |
| **J** | 26.000 | 39.290 | 14.260 | 0.065 | 19.048 | 626.790 | 0.001 | 48.030 | 6067.500 | 99.790 | 86.720 | 100.700 | 26616.000 | 76.668 | 517.439 |
| **2019** |  |  |  |  |  |  |  |  |  |  |  |  |  |  |  |
| **A** | 700.690 | 39.570 | 9.980 | 0.818 | 2438.443 | 9120.860 | 0.070 | 78.780 | 6767.000 | 99.890 | 97.600 | 705.587 | 41850.000 | 58.744 | 2500.033 |
| **B** | 48.850 | 39.730 | 12.080 | 0.148 | 74.899 | 342.380 | 0.006 | 63.490 | 7496.360 | 99.800 | 93.490 | 71.642 | 32504.000 | 90.205 | 1193.438 |
| **C** | 96.730 | 40.960 | 12.420 | 0.249 | 150.421 | 2034.020 | 0.005 | 55.820 | 6002.560 | 99.850 | 97.010 | 107.902 | 34446.000 | 81.012 | 1032.484 |
| **D** | 73.500 | 38.980 | 15.390 | 0.250 | 183.356 | 1906.310 | 0.009 | 54.320 | 7459.420 | 99.990 | 98.930 | 373.453 | 36187.000 | 74.285 | 1029.682 |
| **E** | 68.260 | 39.440 | 14.610 | 0.231 | 218.635 | 1511.490 | 0.007 | 47.730 | 2070.790 | 99.100 | 87.900 | 38.688 | 33674.000 | 68.901 | 1116.760 |
| **F** | 41.000 | 40.760 | 12.530 | 0.035 | 42.234 | 1522.950 | 0.004 | 60.470 | 6690.300 | 99.940 | 93.670 | 115.367 | 34888.000 | 63.304 | 1484.478 |
| **G** | 56.730 | 38.300 | 13.390 | 0.062 | 56.547 | 1332.820 | 0.002 | 49.850 | 4358.100 | 99.570 | 91.760 | 147.313 | 32828.000 | 77.606 | 941.884 |
| **H** | 78.380 | 37.550 | 16.350 | 0.045 | 28.210 | 3898.400 | 0.009 | 60.590 | 3311.730 | 99.870 | 95.700 | 50.366 | 33904.000 | 60.396 | 1854.335 |
| **I** | 45.000 | 39.980 | 8.340 | 0.063 | 69.262 | 1042.710 | 0.001 | 48.630 | 2195.620 | 99.930 | 90.820 | 100.000 | 27016.000 | 68.794 | 722.719 |
| **J** | 26.000 | 36.210 | 14.270 | 0.067 | 14.192 | 727.310 | 0.001 | 46.070 | 6045.000 | 99.790 | 89.710 | 94.739 | 25503.000 | 70.357 | 451.535 |
| **2018** |  |  |  |  |  |  |  |  |  |  |  |  |  |  |  |
| **A** | 701.670 | 38.750 | 9.980 | 0.806 | 2491.106 | 8240.420 | 0.068 | 77.850 | 7037.070 | 99.950 | 97.520 | 692.191 | 38729.100 | 55.699 | 2346.423 |
| **B** | 48.850 | 39.470 | 11.890 | 0.136 | 74.114 | 307.170 | 0.006 | 62.930 | 7514.550 | 99.830 | 93.690 | 67.700 | 29996.000 | 87.573 | 1097.160 |
| **C** | 94.970 | 41.120 | 12.370 | 0.237 | 111.405 | 1947.360 | 0.005 | 54.250 | 5714.650 | 99.860 | 97.420 | 102.652 | 31802.000 | 77.359 | 922.239 |
| **D** | 72.900 | 39.230 | 15.460 | 0.253 | 116.343 | 1850.420 | 0.009 | 52.870 | 2015.960 | 99.770 | 99.150 | 359.012 | 33364.000 | 71.572 | 975.811 |
| **E** | 68.200 | 39.390 | 14.570 | 0.239 | 118.302 | 1427.460 | 0.007 | 45.870 | 2065.920 | 99.100 | 88.640 | 37.024 | 31133.000 | 63.839 | 1023.018 |
| **F** | 41.000 | 40.760 | 12.490 | 0.035 | 35.451 | 1417.200 | 0.004 | 59.190 | 6714.470 | 99.970 | 93.530 | 111.041 | 32226.000 | 59.073 | 1322.004 |
| **G** | 56.370 | 38.430 | 13.030 | 0.063 | 42.520 | 1223.800 | 0.002 | 48.430 | 4326.190 | 99.900 | 91.730 | 141.294 | 30380.000 | 72.435 | 826.293 |
| **H** | 78.380 | 36.240 | 15.770 | 0.039 | 21.579 | 3587.030 | 0.009 | 59.200 | 3525.700 | 99.850 | 95.280 | 47.589 | 31317.000 | 57.286 | 1702.472 |
| **I** | 45.000 | 39.980 | 12.950 | 0.057 | 55.287 | 941.810 | 0.001 | 47.050 | 2191.880 | 99.920 | 90.850 | 90.196 | 24977.000 | 64.859 | 631.565 |
| **J** | 26.000 | 34.000 | 12.370 | 0.068 | 11.528 | 680.600 | 0.001 | 43.830 | 6562.500 | 99.100 | 91.480 | 90.751 | 23491.000 | 66.041 | 394.528 |
| **2017** |  |  |  |  |  |  |  |  |  |  |  |  |  |  |  |
| **A** | 661.080 | 41.110 | 12.050 | 0.748 | 2308.409 | 7172.780 | 0.065 | 77.120 | 6107.000 | 99.940 | 96.980 | 732.099 | 35837.000 | 53.973 | 2173.614 |
| **B** | 48.850 | 38.960 | 11.530 | 0.123 | 63.719 | 277.480 | 0.005 | 62.540 | 7602.000 | 99.840 | 94.180 | 63.028 | 27707.000 | 85.974 | 1025.039 |
| **C** | 93.180 | 41.390 | 12.320 | 0.213 | 91.403 | 1758.630 | 0.004 | 52.830 | 5589.000 | 99.860 | 96.940 | 95.470 | 29402.000 | 74.204 | 800.516 |
| **D** | 71.950 | 39.350 | 15.520 | 0.234 | 102.494 | 1648.700 | 0.008 | 51.560 | 2007.000 | 99.700 | 99.050 | 337.035 | 30874.000 | 69.254 | 896.892 |
| **E** | 66.490 | 38.720 | 13.510 | 0.210 | 87.344 | 1267.900 | 0.006 | 44.150 | 2049.000 | 99.110 | 89.840 | 33.428 | 28757.000 | 57.314 | 899.811 |
| **F** | 41.000 | 40.760 | 10.330 | 0.035 | 17.281 | 1177.120 | 0.004 | 58.070 | 6598.000 | 99.770 | 94.440 | 104.756 | 29849.000 | 56.884 | 1189.185 |
| **G** | 44.290 | 38.710 | 14.280 | 0.053 | 35.827 | 1092.660 | 0.002 | 47.140 | 4013.000 | 99.880 | 91.000 | 132.895 | 28010.000 | 67.593 | 731.747 |
| **H** | 78.380 | 35.800 | 14.340 | 0.037 | 14.736 | 3063.350 | 0.007 | 57.960 | 3566.000 | 99.400 | 95.860 | 54.818 | 28954.000 | 58.017 | 1572.787 |
| **I** | 45.000 | 39.730 | 13.030 | 0.049 | 39.947 | 823.710 | 0.001 | 45.600 | 2169.000 | 99.910 | 91.490 | 87.549 | 23073.000 | 59.354 | 543.946 |
| **J** | 26.000 | 31.810 | 13.210 | 0.060 | 10.921 | 587.240 | 0.001 | 41.740 | 6058.000 | 98.510 | 90.360 | 86.862 | 21678.000 | 63.452 | 353.452 |
| **2016** |  |  |  |  |  |  |  |  |  |  |  |  |  |  |  |
| **A** | 517.740 | 43.150 | 11.870 | 0.514 | 1934.923 | 6199.700 | 0.063 | 76.650 | 8045.000 | 99.930 | 97.420 | 806.796 | 35630.100 | 54.691 | 2154.921 |
| **B** | 48.850 | 38.790 | 11.840 | 0.109 | 110.109 | 247.490 | 0.006 | 62.380 | 7336.000 | 99.810 | 93.690 | 59.247 | 27594.000 | 77.130 | 909.701 |
| **C** | 90.080 | 41.030 | 12.340 | 0.177 | 73.086 | 1551.860 | 0.004 | 51.590 | 5463.000 | 99.860 | 96.760 | 89.404 | 31730.000 | 68.262 | 671.858 |
| **D** | 90.650 | 40.060 | 15.370 | 0.357 | 175.426 | 1726.410 | 0.008 | 50.440 | 2000.000 | 99.760 | 96.520 | 278.142 | 31662.000 | 62.824 | 681.413 |
| **E** | 65.860 | 37.730 | 12.770 | 0.176 | 74.408 | 1137.930 | 0.005 | 42.600 | 2057.000 | 99.080 | 88.970 | 31.712 | 27485.000 | 52.475 | 800.247 |
| **F** | 41.000 | 40.430 | 10.650 | 0.037 | 14.860 | 953.330 | 0.004 | 57.150 | 6368.000 | 99.710 | 94.970 | 100.199 | 30693.000 | 57.939 | 1084.173 |
| **G** | 42.500 | 37.800 | 13.370 | 0.044 | 31.344 | 931.170 | 0.002 | 46.030 | 4402.000 | 99.880 | 92.740 | 125.808 | 25595.000 | 61.617 | 627.201 |
| **H** | 78.380 | 33.650 | 12.370 | 0.034 | 9.447 | 2506.980 | 0.005 | 56.920 | 3519.000 | 99.350 | 94.880 | 49.093 | 29781.000 | 57.729 | 1470.997 |
| **I** | 45.000 | 38.730 | 13.290 | 0.039 | 40.476 | 702.890 | 0.001 | 44.320 | 2125.000 | 99.900 | 90.600 | 81.324 | 25962.000 | 54.120 | 452.312 |
| **J** | 26.000 | 23.120 | 7.060 | 0.048 | 10.396 | 532.430 | 0.001 | 39.830 | 5988.000 | 98.430 | 89.750 | 81.608 | 25468.000 | 55.324 | 306.369 |
| **2015** |  |  |  |  |  |  |  |  |  |  |  |  |  |  |  |
| **A** | 500.590 | 42.620 | 11.740 | 0.511 | 1812.098 | 5740.950 | 0.064 | 75.680 | 7820.000 | 99.930 | 97.330 | 858.603 | 33187.600 | 55.372 | 1996.244 |
| **B** | 44.120 | 43.490 | 11.720 | 0.099 | 72.911 | 244.290 | 0.006 | 61.840 | 7336.000 | 99.850 | 94.270 | 38.551 | 25559.210 | 66.910 | 823.410 |
| **C** | 88.730 | 40.610 | 12.280 | 0.143 | 56.527 | 1449.110 | 0.005 | 50.020 | 5352.000 | 99.860 | 96.600 | 91.142 | 29475.390 | 63.263 | 547.570 |
| **D** | 90.120 | 40.270 | 15.250 | 0.300 | 178.321 | 1633.090 | 0.008 | 49.000 | 2007.000 | 99.750 | 96.280 | 270.477 | 29425.000 | 59.786 | 569.607 |
| **E** | 62.700 | 38.690 | 12.280 | 0.160 | 67.021 | 1107.380 | 0.006 | 40.790 | 2022.000 | 99.110 | 88.560 | 31.335 | 25472.000 | 42.907 | 698.685 |
| **F** | 36.800 | 40.780 | 10.410 | 0.044 | 13.779 | 1075.140 | 0.004 | 55.860 | 6300.000 | 99.750 | 95.440 | 106.939 | 28590.000 | 56.179 | 996.830 |
| **G** | 42.400 | 36.660 | 13.200 | 0.038 | 25.030 | 855.680 | 0.002 | 44.640 | 4346.000 | 99.880 | 92.630 | 129.622 | 23625.000 | 62.381 | 525.720 |
| **H** | 63.600 | 37.670 | 18.670 | 0.032 | 10.948 | 2268.650 | 0.007 | 55.510 | 2227.000 | 99.360 | 95.380 | 48.875 | 27765.000 | 51.844 | 1404.436 |
| **I** | 40.000 | 43.570 | 13.930 | 0.032 | 27.873 | 626.680 | 0.001 | 42.770 | 2028.000 | 99.840 | 90.280 | 81.805 | 23985.000 | 50.996 | 373.061 |
| **J** | 26.000 | 21.960 | 10.400 | 0.040 | 10.030 | 475.680 | 0.002 | 37.690 | 3920.000 | 98.420 | 89.260 | 84.130 | 23509.000 | 52.758 | 265.345 |
| **2014** |  |  |  |  |  |  |  |  |  |  |  |  |  |  |  |
| **A** | 440.000 | 42.500 | 11.600 | 0.584 | 1743.057 | 5385.400 | 0.058 | 74.470 | 8852.000 | 99.940 | 97.410 | 889.792 | 30714.700 | 53.193 | 1774.420 |
| **B** | 44.120 | 43.310 | 11.490 | 0.084 | 81.552 | 256.660 | 0.006 | 61.120 | 7336.000 | 99.850 | 94.460 | 30.188 | 23550.000 | 64.738 | 698.175 |
| **C** | 87.220 | 40.800 | 12.270 | 0.116 | 51.410 | 1345.820 | 0.004 | 48.320 | 5245.000 | 99.870 | 96.670 | 88.943 | 27161.000 | 58.364 | 450.973 |
| **D** | 72.060 | 40.250 | 15.230 | 0.244 | 192.184 | 1517.060 | 0.008 | 47.430 | 12047.000 | 99.740 | 96.140 | 253.948 | 27138.000 | 56.558 | 479.165 |
| **E** | 47.500 | 37.680 | 12.220 | 0.136 | 62.436 | 1107.220 | 0.005 | 38.910 | 2228.000 | 99.030 | 88.340 | 28.902 | 23470.000 | 40.139 | 610.551 |
| **F** | 36.000 | 41.470 | 11.970 | 0.042 | 7.205 | 1278.200 | 0.005 | 54.410 | 7527.000 | 99.770 | 94.940 | 113.595 | 26399.000 | 52.736 | 920.066 |
| **G** | 34.300 | 37.900 | 14.570 | 0.031 | 30.133 | 809.410 | 0.002 | 43.130 | 4595.000 | 99.630 | 91.900 | 125.549 | 21725.000 | 58.769 | 447.507 |
| **H** | 63.000 | 38.960 | 16.600 | 0.038 | 17.225 | 2729.980 | 0.006 | 53.950 | 3437.000 | 99.330 | 95.260 | 47.691 | 25676.000 | 49.421 | 1312.203 |
| **I** | 39.500 | 42.910 | 13.240 | 0.026 | 22.758 | 569.320 | 0.001 | 41.130 | 2016.000 | 99.890 | 89.460 | 80.461 | 22062.000 | 48.502 | 307.220 |
| **J** | 26.000 | 21.850 | 9.880 | 0.032 | 9.510 | 439.220 | 0.002 | 35.510 | 4125.000 | 98.450 | 88.430 | 76.289 | 21613.000 | 48.849 | 250.013 |
| **2013** |  |  |  |  |  |  |  |  |  |  |  |  |  |  |  |
| **A** | 424.000 | 42.200 | 11.200 | 0.508 | 1578.593 | 4777.630 | 0.050 | 73.610 | 9091.000 | 99.940 | 97.290 | 895.620 | 33100.210 | 51.140 | 1508.772 |
| **B** | 44.120 | 44.030 | 11.410 | 0.067 | 57.925 | 257.210 | 0.006 | 60.710 | 7336.000 | 99.910 | 94.650 | 41.259 | 24494.920 | 59.938 | 580.654 |
| **C** | 86.340 | 40.090 | 12.250 | 0.092 | 44.860 | 1264.350 | 0.004 | 46.860 | 5170.000 | 99.820 | 96.550 | 100.352 | 28509.250 | 53.472 | 404.675 |
| **D** | 70.950 | 40.520 | 15.030 | 0.202 | 171.465 | 1369.130 | 0.008 | 46.100 | 12027.000 | 99.730 | 95.640 | 250.010 | 28487.920 | 50.170 | 422.559 |
| **E** | 45.500 | 37.520 | 12.200 | 0.113 | 77.769 | 1033.800 | 0.005 | 37.240 | 2178.000 | 99.010 | 88.000 | 33.013 | 24163.720 | 37.858 | 599.812 |
| **F** | 36.000 | 39.750 | 9.790 | 0.036 | 4.974 | 1239.920 | 0.004 | 53.230 | 9188.000 | 99.830 | 94.700 | 118.840 | 27643.050 | 49.293 | 861.564 |
| **G** | 34.100 | 38.090 | 14.810 | 0.025 | 25.358 | 709.630 | 0.001 | 41.860 | 4522.000 | 99.620 | 90.900 | 127.754 | 22166.740 | 53.882 | 381.225 |
| **H** | 52.000 | 38.960 | 10.550 | 0.043 | 20.863 | 2575.550 | 0.006 | 52.640 | 3193.000 | 99.140 | 94.970 | 47.012 | 26819.500 | 48.773 | 1259.564 |
| **I** | 39.000 | 40.540 | 11.100 | 0.021 | 21.503 | 498.750 | 0.001 | 39.710 | 2010.000 | 99.870 | 88.460 | 77.500 | 22532.650 | 43.669 | 300.527 |
| **J** | 26.000 | 21.150 | 9.430 | 0.026 | 8.896 | 385.600 | 0.001 | 33.560 | 3925.000 | 98.200 | 87.540 | 72.872 | 22257.360 | 45.442 | 246.230 |
| **2012** |  |  |  |  |  |  |  |  |  |  |  |  |  |  |  |
| **A** | 375.000 | 42.000 | 10.810 | 0.420 | 1268.203 | 4189.050 | 0.039 | 72.130 | 9301.000 | 99.970 | 97.260 | 883.260 | 29982.070 | 48.402 | 1284.929 |
| **B** | 44.110 | 42.620 | 10.600 | 0.052 | 58.007 | 216.510 | 0.005 | 59.800 | 7516.000 | 99.580 | 94.380 | 33.317 | 21929.200 | 56.354 | 506.354 |
| **C** | 91.780 | 45.120 | 13.300 | 0.072 | 41.881 | 1123.400 | 0.004 | 45.030 | 5646.000 | 99.890 | 96.470 | 80.646 | 25776.900 | 49.995 | 344.532 |
| **D** | 69.310 | 39.680 | 14.740 | 0.159 | 148.846 | 1138.060 | 0.007 | 44.400 | 11469.000 | 100.000 | 95.550 | 242.654 | 25757.620 | 44.365 | 357.276 |
| **E** | 42.500 | 37.480 | 12.170 | 0.090 | 65.318 | 897.540 | 0.004 | 35.320 | 2079.000 | 97.610 | 88.830 | 32.375 | 21808.420 | 34.698 | 531.421 |
| **F** | 36.000 | 39.000 | 9.580 | 0.028 | 5.489 | 1156.290 | 0.004 | 51.610 | 9299.000 | 98.900 | 94.730 | 130.833 | 24747.580 | 45.581 | 795.770 |
| **G** | 34.000 | 38.000 | 14.960 | 0.020 | 26.945 | 577.970 | 0.001 | 40.260 | 4476.000 | 99.540 | 90.760 | 97.018 | 19827.130 | 48.177 | 317.360 |
| **H** | 50.000 | 36.220 | 10.260 | 0.041 | 12.230 | 2447.550 | 0.006 | 50.910 | 3193.000 | 96.540 | 94.720 | 46.050 | 24139.970 | 46.223 | 1131.026 |
| **I** | 37.500 | 38.400 | 9.830 | 0.016 | 15.490 | 390.010 | 0.001 | 37.980 | 2003.000 | 99.840 | 87.950 | 74.414 | 20299.690 | 38.192 | 257.176 |
| **J** | 20.600 | 26.700 | 9.860 | 0.020 | 7.360 | 309.430 | 0.001 | 31.400 | 3810.000 | 97.900 | 87.810 | 64.467 | 19997.630 | 39.563 | 217.952 |
| **2011** |  |  |  |  |  |  |  |  |  |  |  |  |  |  |  |
| **A** | 342.550 | 41.150 | 10.440 | 0.332 | 986.159 | 3626.280 | 0.032 | 69.900 | 9183.000 | 99.910 | 97.140 | 863.585 | 25981.000 | 46.234 | 1094.070 |
| **B** | 41.110 | 42.520 | 10.010 | 0.038 | 52.456 | 184.050 | 0.005 | 58.280 | 7480.000 | 99.580 | 94.150 | 35.952 | 18775.000 | 55.771 | 510.651 |
| **C** | 95.270 | 36.300 | 14.060 | 0.056 | 40.718 | 951.070 | 0.003 | 42.780 | 5277.000 | 98.920 | 96.410 | 75.383 | 22337.000 | 46.391 | 295.385 |
| **D** | 66.950 | 40.430 | 14.820 | 0.124 | 116.204 | 977.380 | 0.006 | 42.290 | 11332.000 | 99.580 | 95.360 | 215.536 | 22224.000 | 40.583 | 292.538 |
| **E** | 40.500 | 37.460 | 12.130 | 0.070 | 52.420 | 792.890 | 0.003 | 33.100 | 1995.000 | 97.780 | 87.530 | 32.190 | 18768.000 | 30.310 | 454.840 |
| **F** | 36.000 | 37.720 | 9.550 | 0.022 | 2.182 | 996.230 | 0.003 | 49.490 | 8549.000 | 98.880 | 94.190 | 111.268 | 21188.000 | 40.736 | 730.295 |
| **G** | 34.000 | 37.650 | 14.950 | 0.015 | 20.999 | 486.740 | 0.001 | 38.280 | 4403.000 | 98.920 | 90.440 | 61.563 | 17019.000 | 42.757 | 267.757 |
| **H** | 50.000 | 32.840 | 7.690 | 0.032 | 6.786 | 2075.260 | 0.004 | 48.680 | 2941.000 | 96.660 | 94.020 | 40.092 | 20721.000 | 42.700 | 942.142 |
| **I** | 32.500 | 34.580 | 10.590 | 0.013 | 9.500 | 312.650 | 0.001 | 35.920 | 9714.000 | 99.480 | 87.920 | 70.298 | 17365.000 | 32.817 | 215.885 |
| **J** | 13.420 | 39.640 | 11.360 | 0.016 | 5.691 | 261.030 | 0.001 | 29.030 | 3307.000 | 97.760 | 86.570 | 56.662 | 17344.000 | 34.623 | 195.061 |
| **2010** |  |  |  |  |  |  |  |  |  |  |  |  |  |  |  |
| **A** | 326.530 | 40.430 | 9.500 | 0.322 | 833.345 | 3061.820 | 0.024 | 69.000 | 10324.000 | 99.900 | 97.090 | 864.989 | 22243.600 | 46.503 | 919.156 |
| **B** | 38.440 | 42.510 | 9.740 | 0.030 | 36.539 | 150.870 | 0.004 | 57.890 | 7260.000 | 99.570 | 94.050 | 45.952 | 15884.290 | 55.784 | 437.030 |
| **C** | 92.060 | 35.970 | 14.230 | 0.046 | 35.691 | 796.800 | 0.002 | 41.390 | 7517.000 | 99.270 | 96.340 | 74.113 | 18977.710 | 42.785 | 252.443 |
| **D** | 65.000 | 40.110 | 13.430 | 0.103 | 92.039 | 801.780 | 0.004 | 41.020 | 11093.000 | 99.530 | 95.100 | 231.018 | 18913.810 | 36.914 | 237.022 |
| **E** | 40.000 | 37.100 | 11.880 | 0.057 | 32.296 | 621.420 | 0.003 | 31.580 | 2339.000 | 97.380 | 84.890 | 30.624 | 15918.230 | 26.371 | 387.979 |
| **F** | 25.950 | 36.300 | 9.580 | 0.020 | 2.199 | 788.290 | 0.003 | 48.340 | 8162.000 | 98.770 | 94.470 | 110.011 | 17879.970 | 38.923 | 661.155 |
| **G** | 33.200 | 37.650 | 14.090 | 0.011 | 14.028 | 386.890 | 0.001 | 37.080 | 4762.000 | 98.870 | 90.940 | 68.471 | 14509.110 | 38.944 | 233.018 |
| **H** | 40.000 | 32.350 | 7.110 | 0.026 | 7.914 | 1583.780 | 0.003 | 47.410 | 2941.000 | 96.560 | 94.450 | 43.799 | 17544.940 | 39.073 | 760.721 |
| **I** | 30.000 | 34.800 | 10.040 | 0.015 | 7.087 | 245.000 | 0.001 | 34.600 | 11250.000 | 99.620 | 86.610 | 61.871 | 14641.730 | 30.633 | 181.584 |
| **J** | 13.100 | 39.770 | 11.360 | 0.015 | 4.250 | 206.380 | 0.001 | 27.300 | 559.000 | 97.780 | 86.140 | 57.352 | 14811.260 | 28.601 | 172.013 |

**1.2 Below is the standardized index data, with the layout consistent with the previous table.**

Tab.2 Standardized original data

| **City/Year** | **a** | **b** | **c** | **d** | **e** | **f** | **g** | **h** | **i** | **j** | **k** | **l** | **m** | **n** | **o** |
| --- | --- | --- | --- | --- | --- | --- | --- | --- | --- | --- | --- | --- | --- | --- | --- |
| **2022** |  |  |  |  |  |  |  |  |  |  |  |  |  |  |  |
| **A** | 1.000 | 0.951 | 0.427 | 0.977 | 0.985 | 1.000 | 0.974 | 1.000 | 0.653 | 0.968 | 0.895 | 0.709 | 1.000 | 0.471 | 1.000 |
| **B** | 0.045 | 0.796 | 0.426 | 0.190 | 0.046 | 0.029 | 0.061 | 0.710 | 0.638 | 0.942 | 0.666 | 0.058 | 0.702 | 0.966 | 0.449 |
| **C** | 0.132 | 0.935 | 0.685 | 0.307 | 0.062 | 0.214 | 0.054 | 0.614 | 0.467 | 0.957 | 0.589 | 0.106 | 0.763 | 0.856 | 0.422 |
| **D** | 0.082 | 0.771 | 0.898 | 0.343 | 0.142 | 0.205 | 0.127 | 0.580 | 0.595 | 0.997 | 0.272 | 0.412 | 0.827 | 0.675 | 0.323 |
| **E** | 0.070 | 0.844 | 0.713 | 0.190 | 0.077 | 0.147 | 0.078 | 0.460 | 0.132 | 0.740 | 0.000 | 0.017 | 0.738 | 0.644 | 0.424 |
| **F** | 0.073 | 0.840 | 0.483 | 0.017 | 0.008 | 0.168 | 0.051 | 0.663 | 0.157 | 0.983 | 0.503 | 0.111 | 0.779 | 0.555 | 0.596 |
| **G** | 0.068 | 0.846 | 0.730 | 0.077 | 0.026 | 0.133 | 0.014 | 0.487 | 0.329 | 0.876 | 0.226 | 0.130 | 0.716 | 0.813 | 0.390 |
| **H** | 0.082 | 0.737 | 0.796 | 0.036 | 0.014 | 0.552 | 0.250 | 0.669 | 0.275 | 0.962 | 0.790 | 0.076 | 0.762 | 0.477 | 0.771 |
| **I** | 0.040 | 0.872 | 0.434 | 0.061 | 0.025 | 0.086 | 0.008 | 0.478 | 0.145 | 0.980 | 0.310 | 0.099 | 0.515 | 0.680 | 0.284 |
| **J** | 0.016 | 0.989 | 0.683 | 0.084 | 0.008 | 0.057 | 0.008 | 0.434 | 0.494 | 0.939 | 0.301 | 0.089 | 0.455 | 0.700 | 0.153 |
| **2021** |  |  |  |  |  |  |  |  |  |  |  |  |  |  |  |
| **A** | 0.998 | 0.912 | 0.413 | 0.882 | 0.925 | 0.929 | 1.000 | 0.998 | 0.635 | 0.968 | 0.926 | 0.846 | 0.956 | 0.437 | 0.946 |
| **B** | 0.045 | 0.786 | 0.467 | 0.171 | 0.045 | 0.025 | 0.074 | 0.706 | 0.602 | 0.942 | 0.695 | 0.057 | 0.651 | 1.000 | 0.420 |
| **C** | 0.127 | 0.927 | 0.617 | 0.280 | 0.081 | 0.202 | 0.056 | 0.601 | 0.468 | 0.957 | 0.802 | 0.103 | 0.715 | 0.829 | 0.389 |
| **D** | 0.079 | 0.766 | 0.761 | 0.308 | 0.150 | 0.188 | 0.119 | 0.573 | 0.643 | 0.997 | 0.953 | 0.400 | 0.777 | 0.655 | 0.308 |
| **E** | 0.069 | 0.832 | 0.677 | 0.207 | 0.111 | 0.140 | 0.080 | 0.455 | 0.133 | 0.740 | 0.387 | 0.016 | 0.686 | 0.618 | 0.400 |
| **F** | 0.072 | 0.755 | 0.432 | 0.018 | 0.012 | 0.151 | 0.042 | 0.660 | 0.149 | 0.983 | 0.646 | 0.109 | 0.731 | 0.540 | 0.555 |
| **G** | 0.060 | 0.777 | 0.729 | 0.066 | 0.030 | 0.121 | 0.015 | 0.477 | 0.322 | 0.876 | 0.557 | 0.150 | 0.667 | 0.771 | 0.355 |
| **H** | 0.082 | 0.708 | 0.791 | 0.034 | 0.014 | 0.469 | 0.156 | 0.666 | 0.266 | 0.962 | 0.845 | 0.074 | 0.706 | 0.467 | 0.711 |
| **I** | 0.040 | 0.798 | 0.434 | 0.058 | 0.033 | 0.082 | 0.010 | 0.465 | 0.144 | 0.980 | 0.507 | 0.097 | 0.471 | 0.671 | 0.258 |
| **J** | 0.016 | 0.979 | 0.691 | 0.069 | 0.010 | 0.052 | 0.010 | 0.423 | 0.481 | 0.939 | 0.343 | 0.087 | 0.417 | 0.690 | 0.139 |
| **2020** |  |  |  |  |  |  |  |  |  |  |  |  |  |  |  |
| **A** | 0.865 | 0.864 | 0.413 | 1.000 | 1.000 | 0.868 | 0.844 | 0.993 | 0.565 | 0.968 | 0.913 | 0.796 | 0.861 | 0.412 | 0.852 |
| **B** | 0.045 | 0.783 | 0.434 | 0.157 | 0.033 | 0.018 | 0.069 | 0.696 | 0.604 | 0.942 | 0.675 | 0.054 | 0.579 | 0.914 | 0.394 |
| **C** | 0.107 | 0.830 | 0.469 | 0.270 | 0.072 | 0.175 | 0.051 | 0.569 | 0.479 | 0.957 | 0.869 | 0.097 | 0.640 | 0.783 | 0.348 |
| **D** | 0.078 | 0.778 | 0.773 | 0.278 | 0.097 | 0.153 | 0.099 | 0.538 | 0.632 | 0.997 | 0.978 | 0.415 | 0.692 | 0.668 | 0.324 |
| **E** | 0.069 | 0.806 | 0.677 | 0.227 | 0.117 | 0.121 | 0.062 | 0.421 | 0.130 | 0.740 | 0.332 | 0.014 | 0.613 | 0.587 | 0.371 |
| **F** | 0.035 | 0.819 | 0.472 | 0.028 | 0.015 | 0.114 | 0.045 | 0.652 | 0.121 | 0.983 | 0.776 | 0.104 | 0.651 | 0.526 | 0.513 |
| **G** | 0.056 | 0.746 | 0.662 | 0.053 | 0.026 | 0.107 | 0.014 | 0.452 | 0.312 | 0.876 | 0.538 | 0.144 | 0.587 | 0.699 | 0.314 |
| **H** | 0.082 | 0.698 | 0.824 | 0.041 | 0.011 | 0.338 | 0.106 | 0.656 | 0.259 | 0.962 | 0.800 | 0.053 | 0.624 | 0.444 | 0.646 |
| **I** | 0.040 | 0.793 | 0.432 | 0.054 | 0.028 | 0.071 | 0.007 | 0.433 | 0.144 | 0.980 | 0.477 | 0.075 | 0.405 | 0.581 | 0.228 |
| **J** | 0.016 | 0.757 | 0.620 | 0.059 | 0.007 | 0.043 | 0.006 | 0.396 | 0.480 | 0.939 | 0.336 | 0.083 | 0.357 | 0.657 | 0.121 |
| **2019** |  |  |  |  |  |  |  |  |  |  |  |  |  |  |  |
| **A** | 0.865 | 0.768 | 0.252 | 0.885 | 0.939 | 0.815 | 0.819 | 0.985 | 0.540 | 0.968 | 0.917 | 0.781 | 0.806 | 0.423 | 0.817 |
| **B** | 0.045 | 0.775 | 0.432 | 0.150 | 0.028 | 0.017 | 0.067 | 0.692 | 0.604 | 0.942 | 0.698 | 0.049 | 0.531 | 0.834 | 0.359 |
| **C** | 0.105 | 0.826 | 0.462 | 0.261 | 0.057 | 0.171 | 0.051 | 0.545 | 0.474 | 0.957 | 0.886 | 0.091 | 0.588 | 0.714 | 0.302 |
| **D** | 0.076 | 0.744 | 0.717 | 0.262 | 0.070 | 0.159 | 0.096 | 0.517 | 0.601 | 0.997 | 0.988 | 0.398 | 0.639 | 0.626 | 0.301 |
| **E** | 0.069 | 0.763 | 0.650 | 0.241 | 0.083 | 0.124 | 0.071 | 0.391 | 0.132 | 0.740 | 0.399 | 0.011 | 0.565 | 0.556 | 0.332 |
| **F** | 0.035 | 0.818 | 0.471 | 0.026 | 0.015 | 0.125 | 0.043 | 0.634 | 0.534 | 0.983 | 0.707 | 0.100 | 0.601 | 0.483 | 0.461 |
| **G** | 0.055 | 0.715 | 0.545 | 0.055 | 0.021 | 0.107 | 0.014 | 0.431 | 0.331 | 0.876 | 0.605 | 0.137 | 0.540 | 0.669 | 0.270 |
| **H** | 0.082 | 0.684 | 0.800 | 0.037 | 0.010 | 0.340 | 0.106 | 0.637 | 0.240 | 0.962 | 0.816 | 0.025 | 0.572 | 0.445 | 0.591 |
| **I** | 0.040 | 0.786 | 0.110 | 0.056 | 0.026 | 0.081 | 0.007 | 0.408 | 0.142 | 0.980 | 0.555 | 0.082 | 0.369 | 0.554 | 0.193 |
| **J** | 0.016 | 0.628 | 0.621 | 0.061 | 0.005 | 0.052 | 0.006 | 0.359 | 0.478 | 0.939 | 0.496 | 0.076 | 0.324 | 0.575 | 0.098 |
| **2018** |  |  |  |  |  |  |  |  |  |  |  |  |  |  |  |
| **A** | 0.867 | 0.734 | 0.252 | 0.872 | 0.959 | 0.735 | 0.798 | 0.967 | 0.564 | 0.986 | 0.913 | 0.765 | 0.714 | 0.383 | 0.763 |
| **B** | 0.045 | 0.764 | 0.416 | 0.137 | 0.028 | 0.014 | 0.064 | 0.681 | 0.605 | 0.951 | 0.708 | 0.045 | 0.457 | 0.800 | 0.325 |
| **C** | 0.103 | 0.833 | 0.457 | 0.247 | 0.042 | 0.163 | 0.050 | 0.515 | 0.449 | 0.960 | 0.908 | 0.085 | 0.510 | 0.666 | 0.263 |
| **D** | 0.075 | 0.754 | 0.724 | 0.265 | 0.044 | 0.154 | 0.096 | 0.489 | 0.127 | 0.934 | 1.000 | 0.381 | 0.556 | 0.591 | 0.282 |
| **E** | 0.069 | 0.761 | 0.647 | 0.249 | 0.045 | 0.116 | 0.076 | 0.355 | 0.131 | 0.740 | 0.439 | 0.009 | 0.490 | 0.490 | 0.299 |
| **F** | 0.035 | 0.818 | 0.468 | 0.026 | 0.013 | 0.115 | 0.040 | 0.610 | 0.536 | 0.991 | 0.700 | 0.095 | 0.522 | 0.427 | 0.404 |
| **G** | 0.054 | 0.721 | 0.514 | 0.057 | 0.016 | 0.097 | 0.014 | 0.404 | 0.328 | 0.971 | 0.604 | 0.130 | 0.468 | 0.602 | 0.230 |
| **H** | 0.082 | 0.630 | 0.750 | 0.031 | 0.007 | 0.312 | 0.101 | 0.610 | 0.258 | 0.957 | 0.793 | 0.022 | 0.496 | 0.404 | 0.537 |
| **I** | 0.040 | 0.786 | 0.507 | 0.050 | 0.020 | 0.072 | 0.007 | 0.378 | 0.142 | 0.977 | 0.557 | 0.071 | 0.309 | 0.503 | 0.161 |
| **J** | 0.016 | 0.536 | 0.457 | 0.061 | 0.004 | 0.048 | 0.006 | 0.316 | 0.523 | 0.740 | 0.590 | 0.071 | 0.265 | 0.518 | 0.078 |
| **2017** |  |  |  |  |  |  |  |  |  |  |  |  |  |  |  |
| **A** | 0.816 | 0.833 | 0.430 | 0.808 | 0.889 | 0.638 | 0.763 | 0.953 | 0.483 | 0.983 | 0.884 | 0.811 | 0.629 | 0.361 | 0.703 |
| **B** | 0.045 | 0.743 | 0.385 | 0.122 | 0.024 | 0.011 | 0.055 | 0.674 | 0.613 | 0.954 | 0.734 | 0.039 | 0.389 | 0.779 | 0.299 |
| **C** | 0.101 | 0.844 | 0.453 | 0.221 | 0.034 | 0.146 | 0.046 | 0.488 | 0.438 | 0.960 | 0.882 | 0.077 | 0.439 | 0.625 | 0.221 |
| **D** | 0.074 | 0.759 | 0.729 | 0.244 | 0.039 | 0.136 | 0.083 | 0.464 | 0.126 | 0.913 | 0.995 | 0.356 | 0.483 | 0.560 | 0.254 |
| **E** | 0.067 | 0.733 | 0.556 | 0.217 | 0.033 | 0.101 | 0.059 | 0.322 | 0.130 | 0.743 | 0.503 | 0.005 | 0.420 | 0.404 | 0.256 |
| **F** | 0.035 | 0.818 | 0.282 | 0.025 | 0.006 | 0.093 | 0.038 | 0.588 | 0.526 | 0.934 | 0.748 | 0.088 | 0.452 | 0.399 | 0.357 |
| **G** | 0.039 | 0.733 | 0.622 | 0.046 | 0.013 | 0.086 | 0.013 | 0.379 | 0.301 | 0.965 | 0.565 | 0.120 | 0.398 | 0.539 | 0.197 |
| **H** | 0.082 | 0.611 | 0.627 | 0.028 | 0.005 | 0.264 | 0.080 | 0.586 | 0.262 | 0.827 | 0.824 | 0.030 | 0.426 | 0.413 | 0.492 |
| **I** | 0.040 | 0.775 | 0.514 | 0.041 | 0.015 | 0.061 | 0.007 | 0.350 | 0.140 | 0.974 | 0.591 | 0.068 | 0.253 | 0.431 | 0.131 |
| **J** | 0.016 | 0.445 | 0.530 | 0.053 | 0.003 | 0.040 | 0.006 | 0.276 | 0.479 | 0.569 | 0.530 | 0.067 | 0.211 | 0.484 | 0.064 |
| **2016** |  |  |  |  |  |  |  |  |  |  |  |  |  |  |  |
| **A** | 0.635 | 0.918 | 0.414 | 0.551 | 0.745 | 0.549 | 0.747 | 0.944 | 0.652 | 0.980 | 0.908 | 0.898 | 0.623 | 0.370 | 0.696 |
| **B** | 0.045 | 0.736 | 0.412 | 0.107 | 0.042 | 0.009 | 0.058 | 0.671 | 0.590 | 0.945 | 0.708 | 0.035 | 0.386 | 0.663 | 0.259 |
| **C** | 0.097 | 0.829 | 0.455 | 0.181 | 0.027 | 0.127 | 0.042 | 0.465 | 0.427 | 0.960 | 0.872 | 0.070 | 0.508 | 0.547 | 0.175 |
| **D** | 0.098 | 0.789 | 0.716 | 0.380 | 0.067 | 0.143 | 0.088 | 0.443 | 0.125 | 0.931 | 0.859 | 0.288 | 0.506 | 0.476 | 0.179 |
| **E** | 0.066 | 0.692 | 0.492 | 0.180 | 0.028 | 0.090 | 0.052 | 0.293 | 0.130 | 0.734 | 0.456 | 0.003 | 0.383 | 0.341 | 0.221 |
| **F** | 0.035 | 0.804 | 0.309 | 0.028 | 0.005 | 0.073 | 0.034 | 0.571 | 0.506 | 0.916 | 0.777 | 0.082 | 0.477 | 0.412 | 0.320 |
| **G** | 0.037 | 0.695 | 0.543 | 0.036 | 0.011 | 0.071 | 0.013 | 0.358 | 0.335 | 0.965 | 0.658 | 0.112 | 0.327 | 0.461 | 0.160 |
| **H** | 0.082 | 0.521 | 0.457 | 0.025 | 0.003 | 0.214 | 0.057 | 0.566 | 0.258 | 0.812 | 0.772 | 0.023 | 0.450 | 0.410 | 0.456 |
| **I** | 0.040 | 0.733 | 0.537 | 0.031 | 0.015 | 0.050 | 0.008 | 0.325 | 0.136 | 0.971 | 0.543 | 0.060 | 0.338 | 0.363 | 0.098 |
| **J** | 0.016 | 0.082 | 0.000 | 0.041 | 0.003 | 0.035 | 0.010 | 0.240 | 0.473 | 0.546 | 0.498 | 0.061 | 0.323 | 0.378 | 0.047 |
| **2015** |  |  |  |  |  |  |  |  |  |  |  |  |  |  |  |
| **A** | 0.614 | 0.896 | 0.403 | 0.548 | 0.698 | 0.508 | 0.759 | 0.925 | 0.632 | 0.980 | 0.903 | 0.957 | 0.551 | 0.379 | 0.640 |
| **B** | 0.039 | 0.932 | 0.401 | 0.096 | 0.027 | 0.008 | 0.064 | 0.661 | 0.590 | 0.957 | 0.739 | 0.011 | 0.326 | 0.530 | 0.229 |
| **C** | 0.095 | 0.812 | 0.450 | 0.144 | 0.021 | 0.118 | 0.049 | 0.434 | 0.417 | 0.960 | 0.864 | 0.072 | 0.441 | 0.482 | 0.132 |
| **D** | 0.097 | 0.798 | 0.705 | 0.317 | 0.068 | 0.135 | 0.093 | 0.415 | 0.126 | 0.928 | 0.847 | 0.279 | 0.440 | 0.437 | 0.140 |
| **E** | 0.062 | 0.732 | 0.450 | 0.163 | 0.025 | 0.087 | 0.058 | 0.258 | 0.127 | 0.743 | 0.434 | 0.003 | 0.323 | 0.216 | 0.185 |
| **F** | 0.030 | 0.819 | 0.289 | 0.036 | 0.004 | 0.084 | 0.045 | 0.546 | 0.500 | 0.928 | 0.802 | 0.090 | 0.415 | 0.389 | 0.290 |
| **G** | 0.037 | 0.647 | 0.529 | 0.029 | 0.009 | 0.064 | 0.012 | 0.332 | 0.330 | 0.965 | 0.652 | 0.116 | 0.269 | 0.470 | 0.124 |
| **H** | 0.064 | 0.689 | 1.000 | 0.023 | 0.003 | 0.192 | 0.075 | 0.539 | 0.145 | 0.815 | 0.799 | 0.023 | 0.391 | 0.333 | 0.433 |
| **I** | 0.034 | 0.935 | 0.592 | 0.023 | 0.010 | 0.043 | 0.008 | 0.296 | 0.128 | 0.954 | 0.526 | 0.061 | 0.279 | 0.322 | 0.071 |
| **J** | 0.016 | 0.034 | 0.288 | 0.031 | 0.003 | 0.029 | 0.012 | 0.199 | 0.293 | 0.543 | 0.472 | 0.064 | 0.265 | 0.345 | 0.033 |
| **2014** |  |  |  |  |  |  |  |  |  |  |  |  |  |  |  |
| **A** | 0.537 | 0.891 | 0.391 | 0.628 | 0.671 | 0.475 | 0.680 | 0.902 | 0.722 | 0.983 | 0.907 | 0.993 | 0.478 | 0.350 | 0.563 |
| **B** | 0.039 | 0.924 | 0.382 | 0.080 | 0.031 | 0.010 | 0.061 | 0.647 | 0.590 | 0.957 | 0.749 | 0.001 | 0.267 | 0.501 | 0.185 |
| **C** | 0.093 | 0.820 | 0.449 | 0.115 | 0.019 | 0.109 | 0.044 | 0.402 | 0.408 | 0.962 | 0.868 | 0.069 | 0.373 | 0.418 | 0.098 |
| **D** | 0.074 | 0.797 | 0.704 | 0.256 | 0.073 | 0.124 | 0.093 | 0.385 | 1.000 | 0.925 | 0.839 | 0.260 | 0.372 | 0.394 | 0.108 |
| **E** | 0.043 | 0.690 | 0.444 | 0.136 | 0.023 | 0.087 | 0.055 | 0.222 | 0.145 | 0.720 | 0.422 | 0.000 | 0.264 | 0.180 | 0.154 |
| **F** | 0.029 | 0.848 | 0.423 | 0.033 | 0.002 | 0.102 | 0.046 | 0.518 | 0.607 | 0.934 | 0.775 | 0.098 | 0.351 | 0.344 | 0.263 |
| **G** | 0.027 | 0.699 | 0.647 | 0.021 | 0.011 | 0.060 | 0.011 | 0.303 | 0.351 | 0.893 | 0.613 | 0.112 | 0.213 | 0.423 | 0.097 |
| **H** | 0.063 | 0.743 | 0.822 | 0.030 | 0.006 | 0.234 | 0.067 | 0.510 | 0.251 | 0.806 | 0.792 | 0.022 | 0.329 | 0.301 | 0.400 |
| **I** | 0.033 | 0.908 | 0.532 | 0.016 | 0.008 | 0.038 | 0.007 | 0.264 | 0.127 | 0.968 | 0.482 | 0.059 | 0.223 | 0.289 | 0.047 |
| **J** | 0.016 | 0.029 | 0.243 | 0.023 | 0.003 | 0.026 | 0.011 | 0.157 | 0.310 | 0.552 | 0.427 | 0.055 | 0.209 | 0.294 | 0.027 |
| **2013** |  |  |  |  |  |  |  |  |  |  |  |  |  |  |  |
| **A** | 0.517 | 0.878 | 0.357 | 0.545 | 0.608 | 0.420 | 0.584 | 0.886 | 0.743 | 0.983 | 0.901 | 1.000 | 0.548 | 0.324 | 0.469 |
| **B** | 0.039 | 0.955 | 0.375 | 0.061 | 0.021 | 0.010 | 0.067 | 0.639 | 0.590 | 0.974 | 0.760 | 0.014 | 0.294 | 0.439 | 0.143 |
| **C** | 0.092 | 0.790 | 0.447 | 0.089 | 0.016 | 0.101 | 0.040 | 0.374 | 0.401 | 0.948 | 0.861 | 0.082 | 0.413 | 0.354 | 0.082 |
| **D** | 0.073 | 0.808 | 0.686 | 0.209 | 0.065 | 0.111 | 0.084 | 0.360 | 0.998 | 0.922 | 0.812 | 0.255 | 0.412 | 0.311 | 0.088 |
| **E** | 0.041 | 0.683 | 0.443 | 0.111 | 0.029 | 0.080 | 0.052 | 0.190 | 0.141 | 0.714 | 0.404 | 0.005 | 0.285 | 0.150 | 0.150 |
| **F** | 0.029 | 0.776 | 0.235 | 0.027 | 0.001 | 0.099 | 0.043 | 0.496 | 0.751 | 0.951 | 0.762 | 0.104 | 0.387 | 0.299 | 0.242 |
| **G** | 0.026 | 0.707 | 0.668 | 0.015 | 0.009 | 0.051 | 0.008 | 0.278 | 0.345 | 0.890 | 0.559 | 0.114 | 0.226 | 0.359 | 0.073 |
| **H** | 0.049 | 0.743 | 0.301 | 0.034 | 0.007 | 0.220 | 0.065 | 0.485 | 0.229 | 0.751 | 0.777 | 0.021 | 0.363 | 0.293 | 0.382 |
| **I** | 0.033 | 0.809 | 0.348 | 0.010 | 0.007 | 0.032 | 0.006 | 0.237 | 0.126 | 0.962 | 0.429 | 0.056 | 0.237 | 0.226 | 0.045 |
| **J** | 0.016 | 0.000 | 0.204 | 0.016 | 0.003 | 0.021 | 0.008 | 0.120 | 0.293 | 0.480 | 0.380 | 0.051 | 0.229 | 0.249 | 0.026 |
| **2012** |  |  |  |  |  |  |  |  |  |  |  |  |  |  |  |
| **A** | 0.456 | 0.870 | 0.323 | 0.448 | 0.488 | 0.367 | 0.460 | 0.857 | 0.761 | 0.991 | 0.899 | 0.986 | 0.456 | 0.288 | 0.391 |
| **B** | 0.039 | 0.896 | 0.305 | 0.044 | 0.022 | 0.006 | 0.057 | 0.622 | 0.606 | 0.879 | 0.745 | 0.005 | 0.219 | 0.392 | 0.117 |
| **C** | 0.099 | 1.000 | 0.537 | 0.067 | 0.015 | 0.088 | 0.036 | 0.339 | 0.443 | 0.968 | 0.857 | 0.060 | 0.332 | 0.309 | 0.061 |
| **D** | 0.071 | 0.773 | 0.661 | 0.161 | 0.057 | 0.090 | 0.074 | 0.327 | 0.950 | 1.000 | 0.808 | 0.247 | 0.332 | 0.235 | 0.065 |
| **E** | 0.037 | 0.681 | 0.440 | 0.086 | 0.024 | 0.068 | 0.043 | 0.153 | 0.132 | 0.309 | 0.449 | 0.004 | 0.215 | 0.109 | 0.126 |
| **F** | 0.029 | 0.745 | 0.217 | 0.018 | 0.001 | 0.091 | 0.038 | 0.465 | 0.761 | 0.682 | 0.764 | 0.118 | 0.302 | 0.251 | 0.219 |
| **G** | 0.026 | 0.703 | 0.680 | 0.009 | 0.010 | 0.039 | 0.006 | 0.248 | 0.341 | 0.867 | 0.552 | 0.079 | 0.157 | 0.285 | 0.051 |
| **H** | 0.046 | 0.629 | 0.276 | 0.033 | 0.004 | 0.209 | 0.062 | 0.452 | 0.229 | 0.000 | 0.763 | 0.020 | 0.284 | 0.259 | 0.337 |
| **I** | 0.031 | 0.720 | 0.239 | 0.005 | 0.005 | 0.022 | 0.004 | 0.204 | 0.126 | 0.954 | 0.402 | 0.053 | 0.171 | 0.154 | 0.030 |
| **J** | 0.009 | 0.232 | 0.241 | 0.010 | 0.002 | 0.014 | 0.006 | 0.078 | 0.283 | 0.393 | 0.394 | 0.041 | 0.162 | 0.172 | 0.016 |
| **2011** |  |  |  |  |  |  |  |  |  |  |  |  |  |  |  |
| **A** | 0.415 | 0.834 | 0.291 | 0.351 | 0.379 | 0.316 | 0.367 | 0.815 | 0.751 | 0.974 | 0.893 | 0.963 | 0.338 | 0.260 | 0.324 |
| **B** | 0.035 | 0.892 | 0.254 | 0.029 | 0.019 | 0.003 | 0.051 | 0.592 | 0.602 | 0.879 | 0.733 | 0.008 | 0.126 | 0.384 | 0.119 |
| **C** | 0.103 | 0.632 | 0.603 | 0.048 | 0.015 | 0.073 | 0.026 | 0.296 | 0.411 | 0.688 | 0.854 | 0.054 | 0.231 | 0.262 | 0.043 |
| **D** | 0.068 | 0.804 | 0.668 | 0.123 | 0.044 | 0.075 | 0.061 | 0.287 | 0.938 | 0.879 | 0.798 | 0.215 | 0.228 | 0.186 | 0.042 |
| **E** | 0.034 | 0.680 | 0.437 | 0.064 | 0.019 | 0.058 | 0.033 | 0.111 | 0.125 | 0.358 | 0.379 | 0.004 | 0.126 | 0.051 | 0.099 |
| **F** | 0.029 | 0.691 | 0.214 | 0.012 | 0.000 | 0.077 | 0.032 | 0.424 | 0.696 | 0.676 | 0.735 | 0.095 | 0.197 | 0.188 | 0.196 |
| **G** | 0.026 | 0.688 | 0.680 | 0.004 | 0.007 | 0.031 | 0.004 | 0.210 | 0.335 | 0.688 | 0.535 | 0.038 | 0.074 | 0.214 | 0.034 |
| **H** | 0.046 | 0.488 | 0.054 | 0.023 | 0.002 | 0.175 | 0.043 | 0.409 | 0.207 | 0.035 | 0.726 | 0.013 | 0.183 | 0.213 | 0.270 |
| **I** | 0.024 | 0.560 | 0.304 | 0.002 | 0.003 | 0.015 | 0.001 | 0.165 | 0.797 | 0.850 | 0.400 | 0.048 | 0.084 | 0.084 | 0.015 |
| **J** | 0.000 | 0.771 | 0.370 | 0.005 | 0.001 | 0.010 | 0.004 | 0.033 | 0.239 | 0.353 | 0.328 | 0.032 | 0.084 | 0.108 | 0.008 |
| **2010** |  |  |  |  |  |  |  |  |  |  |  |  |  |  |  |
| **A** | 0.395 | 0.804 | 0.210 | 0.340 | 0.320 | 0.264 | 0.277 | 0.797 | 0.850 | 0.971 | 0.890 | 0.965 | 0.228 | 0.263 | 0.262 |
| **B** | 0.032 | 0.891 | 0.231 | 0.021 | 0.013 | 0.000 | 0.034 | 0.585 | 0.583 | 0.876 | 0.728 | 0.020 | 0.041 | 0.384 | 0.093 |
| **C** | 0.099 | 0.618 | 0.618 | 0.038 | 0.013 | 0.059 | 0.018 | 0.269 | 0.606 | 0.789 | 0.850 | 0.052 | 0.132 | 0.214 | 0.028 |
| **D** | 0.065 | 0.791 | 0.549 | 0.100 | 0.035 | 0.059 | 0.044 | 0.262 | 0.917 | 0.864 | 0.784 | 0.233 | 0.130 | 0.138 | 0.023 |
| **E** | 0.034 | 0.665 | 0.415 | 0.050 | 0.012 | 0.043 | 0.024 | 0.082 | 0.155 | 0.243 | 0.238 | 0.002 | 0.042 | 0.000 | 0.076 |
| **F** | 0.016 | 0.632 | 0.217 | 0.009 | 0.000 | 0.058 | 0.026 | 0.402 | 0.662 | 0.645 | 0.750 | 0.094 | 0.099 | 0.164 | 0.172 |
| **G** | 0.025 | 0.688 | 0.606 | 0.000 | 0.005 | 0.021 | 0.001 | 0.187 | 0.366 | 0.673 | 0.561 | 0.046 | 0.000 | 0.164 | 0.021 |
| **H** | 0.034 | 0.467 | 0.004 | 0.016 | 0.002 | 0.130 | 0.027 | 0.385 | 0.207 | 0.006 | 0.749 | 0.017 | 0.090 | 0.166 | 0.207 |
| **I** | 0.021 | 0.569 | 0.257 | 0.004 | 0.002 | 0.009 | 0.000 | 0.140 | 0.931 | 0.890 | 0.330 | 0.038 | 0.004 | 0.056 | 0.003 |
| **J** | 0.000 | 0.777 | 0.370 | 0.004 | 0.001 | 0.005 | 0.000 | 0.000 | 0.000 | 0.358 | 0.305 | 0.033 | 0.009 | 0.029 | 0.000 |

**1.3 According to the entropy method calculation formula, the information entropy value (e), the information utility value (d), and the weight coefficient (w) are obtained respectively.**

Tab.3 The weight values of the urbanization of population indicator system and the related calculation process values

| **Index layer** | **Information entropy value (e)** | **Information utility value (d)** | **Weight coefficient (w)** |
| --- | --- | --- | --- |
| Urban built-up area | 0.8548 | 0.1452 | 18.38% |
| Green coverage rate of built-up areas | 0.9925 | 0.0075 | 0.94% |
| Per capita park and green space area | 0.9835 | 0.0165 | 2.08% |
| Fixed asset investment per unit area | 0.8805 | 0.1195 | 15.13% |
| Real estate investment per unit area | 0.7662 | 0.2338 | 29.61% |
| Value of secondary and tertiary industries per unit area | 0.9044 | 0.0956 | 12.11% |
| Fiscal revenue per unit area | 0.8282 | 0.1718 | 21.75% |

Tab.4 The weight values of the urbanization of land indicator system and the related calculation process values

| **Index layer** | **Information entropy value (e)** | **Information utility value (d)** | **Weight coefficient (w)** |
| --- | --- | --- | --- |
| Proportion of non-agricultural population | 0.9778 | 0.0222 | 6.88% |
| Population density | 0.9672 | 0.0328 | 10.15% |
| Proportion of employees in the secondary and tertiary industries in urban areas | 0.9915 | 0.0085 | 2.63% |
| Proportion of value of the secondary and tertiary industries in GDP | 0.9890 | 0.0110 | 3.40% |
| Number of higher education students per 10,000 people | 0.8601 | 0.1399 | 43.28% |
| Per capita disposable income of urban residents | 0.9716 | 0.0284 | 8.79% |
| Number of hospital beds per 10,000 people | 0.9764 | 0.0236 | 7.30% |
| Number of private cars per 10,000 urban households | 0.9432 | 0.0568 | 17.58% |

**1.4 Calculate comprehensive weight**

The study suggests that land urbanization and population urbanization are two inseparable components in the process of "new urbanization". They are equally important to the development of "new urbanization", hence their comprehensive weight values are both assigned 0.500. The comprehensive weight values of each indicator are calculated, as shown in Table 5.

Tab.5 The weight of each index of new urbanization in Shaanxi Province

| **Objective layer** | **Criterion layer** | **Index layer** | **Comprehensive weight** |
| --- | --- | --- | --- |
| Land  urbanization  (0.500) | Land structure  (0.107) | Urban built-up area | 0.092 |
|  |  | Green coverage rate of built-up areas | 0.005 |
|  |  | Per capita park and green space area | 0.010 |
|  | Land input  (0.224) | Fixed asset investment per unit area | 0.076 |
|  |  | Real estate investment per unit area | 0.148 |
|  | Land output  (0.169) | Value of secondary and tertiary industries per unit area | 0.061 |
|  |  | Fiscal revenue per unit area | 0.109 |
| Population urbanization  (0.500) | Demographic  Structure  (0.085) | Proportion of non-agricultural population | 0.034 |
|  |  | Population density | 0.051 |
|  | Industrial  Structure  (0.030) | Proportion of employees in the secondary and tertiary industries in urban areas | 0.013 |
|  |  | Proportion of value of the secondary and tertiary industries in GDP | 0.017 |
|  | Life quality of residents  (0.260) | Number of higher education students per 10,000 people | 0.216 |
|  |  | Per capita disposable income of urban residents | 0.044 |
|  | Lifestyle of residents  (0.124) | Number of hospital beds per 10,000 people | 0.037 |
|  |  | Number of private cars per 10,000 urban households | 0.088 |

**2. Original data of the indicators and the calculation process of the entropy weight method**

Tab. 6 The calculated result of coupling coordination degree

| **City/Year** | **Coupling degree (C)** | **Coordination degree (T)** | **Coupling coordination degree (D)** | **Levels** |
| --- | --- | --- | --- | --- |
| **2022** |  |  |  |  |
| **A** | 0.995 | 0.895 | 0.944 | 10 |
| **B** | 0.780 | 0.251 | 0.442 | 5 |
| **C** | 0.906 | 0.277 | 0.501 | 6 |
| **D** | 0.900 | 0.350 | 0.561 | 6 |
| **E** | 0.941 | 0.200 | 0.434 | 5 |
| **F** | 0.754 | 0.227 | 0.414 | 5 |
| **G** | 0.787 | 0.219 | 0.416 | 5 |
| **H** | 0.922 | 0.293 | 0.520 | 6 |
| **I** | 0.775 | 0.173 | 0.366 | 4 |
| **J** | 0.763 | 0.171 | 0.361 | 4 |
| **2021** |  |  |  |  |
| **A** | 0.998 | 0.898 | 0.947 | 10 |
| **B** | 0.786 | 0.245 | 0.439 | 5 |
| **C** | 0.907 | 0.273 | 0.497 | 5 |
| **D** | 0.884 | 0.352 | 0.558 | 6 |
| **E** | 0.952 | 0.207 | 0.443 | 5 |
| **F** | 0.745 | 0.220 | 0.405 | 5 |
| **G** | 0.773 | 0.220 | 0.412 | 5 |
| **H** | 0.893 | 0.269 | 0.490 | 5 |
| **I** | 0.782 | 0.171 | 0.366 | 4 |
| **J** | 0.762 | 0.166 | 0.356 | 4 |
| **2020** |  |  |  |  |
| **A** | 0.997 | 0.857 | 0.924 | 10 |
| **B** | 0.776 | 0.231 | 0.424 | 5 |
| **C** | 0.895 | 0.255 | 0.478 | 5 |
| **D** | 0.848 | 0.338 | 0.535 | 6 |
| **E** | 0.960 | 0.196 | 0.434 | 5 |
| **F** | 0.732 | 0.208 | 0.390 | 4 |
| **G** | 0.767 | 0.204 | 0.395 | 4 |
| **H** | 0.872 | 0.240 | 0.457 | 5 |
| **I** | 0.795 | 0.154 | 0.350 | 4 |
| **J** | 0.739 | 0.154 | 0.338 | 4 |
| **2019** |  |  |  |  |
| **A** | 0.998 | 0.821 | 0.906 | 10 |
| **B** | 0.778 | 0.221 | 0.414 | 5 |
| **C** | 0.898 | 0.241 | 0.465 | 5 |
| **D** | 0.840 | 0.320 | 0.518 | 6 |
| **E** | 0.962 | 0.186 | 0.423 | 5 |
| **F** | 0.720 | 0.218 | 0.397 | 4 |
| **G** | 0.764 | 0.194 | 0.385 | 4 |
| **H** | 0.887 | 0.225 | 0.446 | 5 |
| **I** | 0.778 | 0.147 | 0.338 | 4 |
| **J** | 0.750 | 0.148 | 0.333 | 4 |
| **2018** |  |  |  |  |
| **A** | 0.998 | 0.803 | 0.895 | 9 |
| **B** | 0.780 | 0.211 | 0.405 | 5 |
| **C** | 0.900 | 0.225 | 0.450 | 5 |
| **D** | 0.863 | 0.280 | 0.492 | 5 |
| **E** | 0.963 | 0.171 | 0.406 | 5 |
| **F** | 0.728 | 0.205 | 0.386 | 4 |
| **G** | 0.769 | 0.182 | 0.374 | 4 |
| **H** | 0.887 | 0.210 | 0.432 | 5 |
| **I** | 0.820 | 0.139 | 0.337 | 4 |
| **J** | 0.740 | 0.138 | 0.319 | 4 |
| **2017** |  |  |  |  |
| **A** | 0.999 | 0.771 | 0.878 | 9 |
| **B** | 0.768 | 0.201 | 0.393 | 4 |
| **C** | 0.899 | 0.208 | 0.433 | 5 |
| **D** | 0.862 | 0.262 | 0.475 | 5 |
| **E** | 0.958 | 0.152 | 0.382 | 4 |
| **F** | 0.706 | 0.189 | 0.365 | 4 |
| **G** | 0.774 | 0.166 | 0.359 | 4 |
| **H** | 0.868 | 0.196 | 0.413 | 5 |
| **I** | 0.822 | 0.127 | 0.324 | 4 |
| **J** | 0.760 | 0.124 | 0.307 | 4 |
| **2016** |  |  |  |  |
| **A** | 0.997 | 0.734 | 0.855 | 9 |
| **B** | 0.797 | 0.192 | 0.391 | 4 |
| **C** | 0.887 | 0.195 | 0.416 | 5 |
| **D** | 0.936 | 0.253 | 0.487 | 5 |
| **E** | 0.956 | 0.137 | 0.362 | 4 |
| **F** | 0.700 | 0.184 | 0.359 | 4 |
| **G** | 0.763 | 0.155 | 0.344 | 4 |
| **H** | 0.837 | 0.182 | 0.391 | 4 |
| **I** | 0.823 | 0.121 | 0.316 | 4 |
| **J** | 0.653 | 0.112 | 0.270 | 3 |
| **2015** |  |  |  |  |
| **A** | 0.995 | 0.726 | 0.850 | 9 |
| **B** | 0.808 | 0.175 | 0.376 | 4 |
| **C** | 0.887 | 0.181 | 0.401 | 5 |
| **D** | 0.937 | 0.237 | 0.472 | 5 |
| **E** | 0.970 | 0.123 | 0.345 | 4 |
| **F** | 0.724 | 0.180 | 0.361 | 4 |
| **G** | 0.758 | 0.148 | 0.334 | 4 |
| **H** | 0.885 | 0.174 | 0.393 | 4 |
| **I** | 0.838 | 0.112 | 0.306 | 4 |
| **J** | 0.729 | 0.098 | 0.268 | 3 |
| **2014** |  |  |  |  |
| **A** | 0.993 | 0.711 | 0.840 | 9 |
| **B** | 0.817 | 0.164 | 0.366 | 4 |
| **C** | 0.887 | 0.167 | 0.385 | 4 |
| **D** | 0.887 | 0.263 | 0.483 | 5 |
| **E** | 0.971 | 0.110 | 0.327 | 4 |
| **F** | 0.744 | 0.181 | 0.367 | 4 |
| **G** | 0.775 | 0.138 | 0.328 | 4 |
| **H** | 0.893 | 0.172 | 0.392 | 4 |
| **I** | 0.839 | 0.102 | 0.293 | 3 |
| **J** | 0.730 | 0.089 | 0.255 | 3 |
| **2013** |  |  |  |  |
| **A** | 0.986 | 0.675 | 0.816 | 9 |
| **B** | 0.806 | 0.160 | 0.359 | 4 |
| **C** | 0.872 | 0.163 | 0.377 | 4 |
| **D** | 0.872 | 0.250 | 0.467 | 5 |
| **E** | 0.967 | 0.107 | 0.322 | 4 |
| **F** | 0.703 | 0.184 | 0.360 | 4 |
| **G** | 0.774 | 0.132 | 0.319 | 4 |
| **H** | 0.865 | 0.161 | 0.374 | 4 |
| **I** | 0.816 | 0.094 | 0.277 | 3 |
| **J** | 0.718 | 0.082 | 0.242 | 3 |
| **2012** |  |  |  |  |
| **A** | 0.971 | 0.612 | 0.771 | 8 |
| **B** | 0.800 | 0.146 | 0.341 | 4 |
| **C** | 0.888 | 0.151 | 0.367 | 4 |
| **D** | 0.859 | 0.230 | 0.444 | 5 |
| **E** | 0.977 | 0.089 | 0.295 | 3 |
| **F** | 0.698 | 0.173 | 0.348 | 4 |
| **G** | 0.803 | 0.113 | 0.302 | 4 |
| **H** | 0.889 | 0.138 | 0.351 | 4 |
| **I** | 0.805 | 0.081 | 0.256 | 3 |
| **J** | 0.756 | 0.070 | 0.229 | 3 |
| **2011** |  |  |  |  |
| **A** | 0.952 | 0.552 | 0.725 | 8 |
| **B** | 0.782 | 0.138 | 0.328 | 4 |
| **C** | 0.898 | 0.132 | 0.344 | 4 |
| **D** | 0.855 | 0.204 | 0.417 | 5 |
| **E** | 0.986 | 0.074 | 0.270 | 3 |
| **F** | 0.706 | 0.152 | 0.327 | 4 |
| **G** | 0.848 | 0.091 | 0.278 | 3 |
| **H** | 0.874 | 0.114 | 0.316 | 4 |
| **I** | 0.700 | 0.102 | 0.267 | 3 |
| **J** | 0.859 | 0.058 | 0.223 | 3 |
| **2010** |  |  |  |  |
| **A** | 0.932 | 0.521 | 0.697 | 7 |
| **B** | 0.750 | 0.129 | 0.311 | 4 |
| **C** | 0.875 | 0.131 | 0.338 | 4 |
| **D** | 0.827 | 0.190 | 0.396 | 4 |
| **E** | 0.995 | 0.058 | 0.240 | 3 |
| **F** | 0.685 | 0.138 | 0.308 | 4 |
| **G** | 0.833 | 0.085 | 0.266 | 3 |
| **H** | 0.850 | 0.096 | 0.286 | 3 |
| **I** | 0.686 | 0.099 | 0.260 | 3 |
| **J** | 0.960 | 0.037 | 0.188 | 2 |

**3. The calculation process of ANP model**

Tab.7 The indicator system for the problems caused by the lagging land urbanization in Shaanxi Province

| **Objective layer** | **Criterion layer** | **Network layer** |
| --- | --- | --- |
| Effects or problems caused by the lag of land urbanization  (A) | Limits to urban re-expansion  (B_1_) | Scarcity of land resources (B_11_) |
|  |  | Irrationality of land planning (B_12_) |
|  | Inadequate of infrastructure support  (B_2_) | Undersupply of infrastructure (B_21_) |
|  |  | Maldistribution of infrastructure (B_22_) |
|  | Increasing social inequality  (B_3_) | Widening gap between rich and poor (B_31_) |
|  |  | Social housing inequality (B_32_) |
|  | Damage to the ecological environment  (B_4_) | Destruction of ecosystem (B_41_) |
|  |  | Disturbance of ecological balance (B_42_) |
|  |  | Weakening of ecological service function (B_43_) |

**3.1 Construction of the association relation table**

Tab.8 The correlation table of the indicators of the problems caused by the lagging land urbanization in Shaanxi Province

|  | | B_1_ | | B_2_ | | B_3_ | | B_4_ | | |
| --- | --- | --- | --- | --- | --- | --- | --- | --- | --- | --- |
|  |  | B_11_ | B_12_ | B_21_ | B_22_ | B_31_ | B_32_ | B_41_ | B_42_ | B_43_ |
| B_1_ | B_11_ |  | √ | √ | √ |  |  |  |  | √ |
|  | B_12_ |  |  | √ | √ |  | √ |  |  | √ |
| B_2_ | B_21_ | √ | √ |  |  |  | √ |  |  |  |
|  | B_22_ |  | √ |  |  | √ | √ |  |  | √ |
| B_3_ | B_31_ | √ | √ |  |  |  | √ |  |  |  |
|  | B_32_ | √ | √ | √ | √ |  |  |  |  |  |
| B_4_ | B_41_ | √ | √ |  |  |  |  |  | √ | √ |
|  | B_42_ | √ | √ | √ |  |  |  | √ |  | √ |
|  | B_43_ | √ | √ | √ |  |  |  | √ | √ | √ |

“√” represents that the element on the row corresponding to this cell can affect the element on the column.

**3.2 Evaluation model construction**


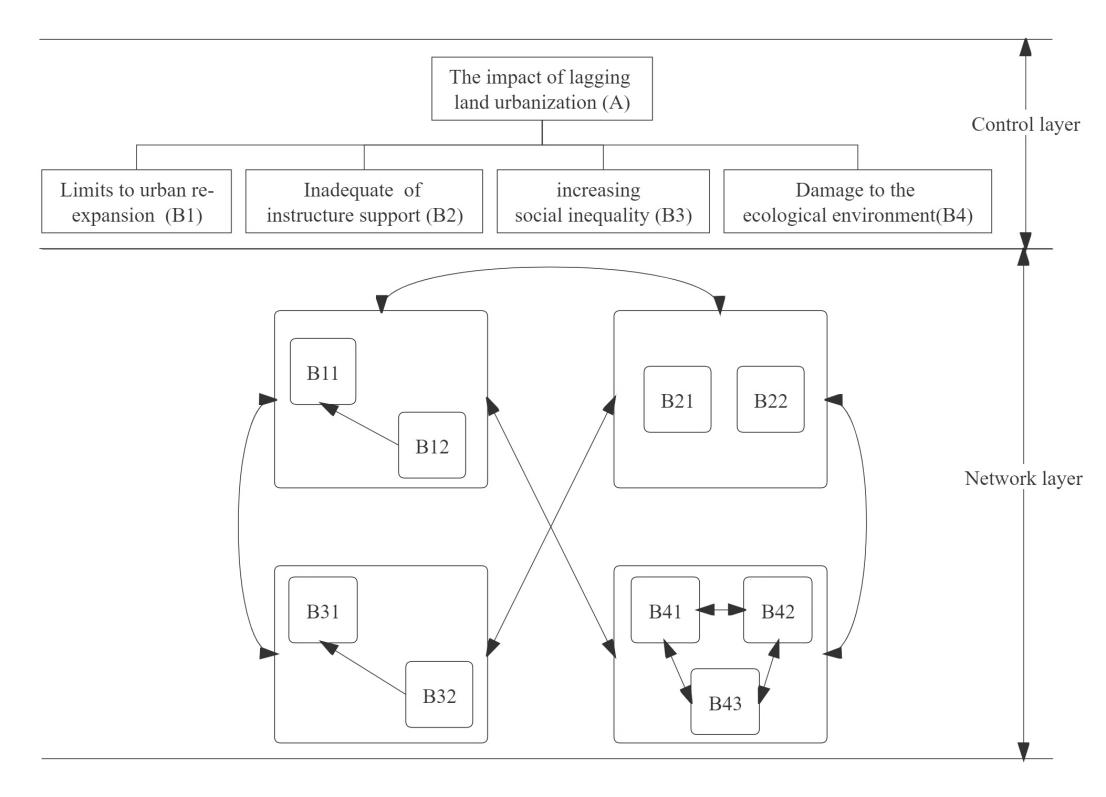


Fig.1 Evaluation model of the impacts or problems caused by the lagging land urbanization in Shaanxi Province.

**3.3 Construct and assign values to pairwise judgment matrices**

Constructing pairwise judgment matrices, the 1-9 scale method is utilized to measure the dominance among elements, and the results of element weights and consistency tests are obtained.

Tab.9 The pairwise comparison matrix concerning the Limits to urban re-expansion

|  | B_1_ | B_2_ | B_3_ | B_4_ | weight |
| --- | --- | --- | --- | --- | --- |
| B_1_ | 1 | 2 | 4 | 3 | 0.467 |
| B_2_ | 1/2 | 1 | 3 | 2 | 0.277 |
| B_3_ | 1/4 | 1/3 | 1 | 1/2 | 0.095 |
| B_4_ | 1/3 | 1/2 | 2 | 1 | 0.160 |
| C.R.=0.012 | | | | | |

Tab.10 The pairwise comparison matrix concerning the Inadequate of infrastructure support

|  | B_1_ | B_3_ | B_4_ | weight |
| --- | --- | --- | --- | --- |
| B_1_ | 1 | 1 | 2 | 0.413 |
| B_3_ | 1 | 1 | 1 | 0.327 |
| B_4_ | 1/2 | 1 | 1 | 0.260 |
| C.R.=0.052 | | | | |

Tab.11 The pairwise comparison matrix concerning the Increasing social inequality

|  | B_1_ | B_2_ | B_3_ | weight |
| --- | --- | --- | --- | --- |
| B_1_ | 1 | 2 | 2 | 0.493 |
| B_2_ | 1/2 | 1 | 2 | 0.311 |
| B_3_ | 1/2 | 1/2 | 1 | 0.196 |
| C.R.=0.052 | | | | |

Tab.12 The pairwise comparison matrix concerning the Damage to the ecological environment

|  | B_1_ | B_2_ | B_4_ | weight |
| --- | --- | --- | --- | --- |
| B_1_ | 1 | 3 | 2 | 0.550 |
| B_2_ | 1/3 | 1 | 2 | 0.210 |
| B_4_ | 1/2 | 1/2 | 1 | 0.240 |
| C.R.=0.018 | | | | |

**3.4 Calculate the supermatrix**

Due to the complexity and difficulty of the ANP calculation, the study utilizes Super Decision (v3.2) for the calculations. This process includes calculating the unweighted supermatrix, the weighted supermatrix, and the limit supermatrix.

Tab.13 Unweighted supermatrix

|  | **B_11_** | **B_12_** | **B_21_** | **B_22_** | **B_31_** | **B_32_** | **B_41_** | **B_42_** | **B_43_** |
| --- | --- | --- | --- | --- | --- | --- | --- | --- | --- |
| B_11_ | 0.000 | 0.000 | 0.800 | 0.000 | 0.250 | 0.200 | 0.200 | 0.250 | 0.250 |
| B_12_ | 1.000 | 0.000 | 0.200 | 1.000 | 0.750 | 0.800 | 0.800 | 0.750 | 0.750 |
| B_21_ | 0.667 | 0.750 | 0.000 | 0.000 | 0.000 | 0.167 | 0.000 | 1.000 | 1.000 |
| B_22_ | 0.333 | 0.250 | 0.000 | 0.000 | 0.000 | 0.833 | 0.000 | 0.000 | 0.000 |
| B_31_ | 0.000 | 0.000 | 0.000 | 0.200 | 0.000 | 0.000 | 0.000 | 0.000 | 0.000 |
| B_32_ | 0.000 | 1.000 | 1.000 | 0.800 | 1.000 | 0.000 | 0.000 | 0.000 | 0.000 |
| B_41_ | 0.000 | 0.000 | 0.000 | 0.000 | 0.000 | 0.000 | 0.000 | 0.750 | 0.614 |
| B_42_ | 0.000 | 0.000 | 0.000 | 0.000 | 0.000 | 0.000 | 0.800 | 0.000 | 0.117 |
| B_43_ | 1.000 | 1.000 | 0.000 | 1.000 | 0.000 | 0.000 | 0.200 | 0.250 | 0.268 |

Tab.14 Weighted supermatrix

|  | **B_11_** | **B_12_** | **B_21_** | **B_22_** | **B_31_** | **B_32_** | **B_41_** | **B_42_** | **B_43_** |
| --- | --- | --- | --- | --- | --- | --- | --- | --- | --- |
| B_11_ | 0.000 | 0.000 | 0.446 | 0.000 | 0.179 | 0.123 | 0.139 | 0.137 | 0.137 |
| B_12_ | 0.517 | 0.000 | 0.111 | 0.413 | 0.537 | 0.491 | 0.557 | 0.412 | 0.412 |
| B_21_ | 0.204 | 0.390 | 0.000 | 0.000 | 0.000 | 0.064 | 0.000 | 0.210 | 0.210 |
| B_22_ | 0.102 | 0.130 | 0.000 | 0.000 | 0.000 | 0.322 | 0.000 | 0.000 | 0.000 |
| B_31_ | 0.000 | 0.000 | 0.000 | 0.066 | 0.000 | 0.000 | 0.000 | 0.000 | 0.000 |
| B_32_ | 0.000 | 0.179 | 0.442 | 0.262 | 0.284 | 0.000 | 0.000 | 0.000 | 0.000 |
| B_41_ | 0.000 | 0.000 | 0.000 | 0.000 | 0.000 | 0.000 | 0.000 | 0.180 | 0.148 |
| B_42_ | 0.000 | 0.000 | 0.000 | 0.000 | 0.000 | 0.000 | 0.243 | 0.000 | 0.028 |
| B_43_ | 0.177 | 0.301 | 0.000 | 0.260 | 0.000 | 0.000 | 0.061 | 0.060 | 0.064 |

Tab.15 Limit supermatrix

|  | **B_11_** | **B_12_** | **B_21_** | **B_22_** | **B_31_** | **B_32_** | **B_41_** | **B_42_** | **B_43_** |
| --- | --- | --- | --- | --- | --- | --- | --- | --- | --- |
| B_11_ | 0.121 | 0.121 | 0.121 | 0.121 | 0.121 | 0.121 | 0.121 | 0.121 | 0.121 |
| B_12_ | 0.275 | 0.275 | 0.275 | 0.275 | 0.275 | 0.275 | 0.275 | 0.275 | 0.275 |
| B_21_ | 0.174 | 0.174 | 0.174 | 0.174 | 0.174 | 0.174 | 0.174 | 0.174 | 0.174 |
| B_22_ | 0.098 | 0.098 | 0.098 | 0.098 | 0.098 | 0.098 | 0.098 | 0.098 | 0.098 |
| B_31_ | 0.006 | 0.006 | 0.006 | 0.006 | 0.006 | 0.006 | 0.006 | 0.006 | 0.006 |
| B_32_ | 0.154 | 0.154 | 0.154 | 0.154 | 0.154 | 0.154 | 0.154 | 0.154 | 0.154 |
| B_41_ | 0.022 | 0.022 | 0.022 | 0.022 | 0.022 | 0.022 | 0.022 | 0.022 | 0.022 |
| B_42_ | 0.009 | 0.009 | 0.009 | 0.009 | 0.009 | 0.009 | 0.009 | 0.009 | 0.009 |
| B_43_ | 0.141 | 0.141 | 0.141 | 0.141 | 0.141 | 0.141 | 0.141 | 0.141 | 0.141 |

**3.5 The weights of various factors resulting from the lag in land urbanization**

Tab.16 The weights of various factors resulting from the lag in land urbanization

| **Criterion layer** | **Weight** | **Network layer** | **Comprehensive weight** |
| --- | --- | --- | --- |
| Limits to urban re-expansion  (B_1_) | 0.396 | Scarcity of land resources (B_11_) | 0.121 |
|  |  | Irrationality of land planning (B_12_) | 0.275 |
| Inadequate of infrastructure support  (B_2_) | 0.272 | Undersupply of infrastructure (B_21_) | 0.174 |
|  |  | Maldistribution of infrastructure (B_22_) | 0.098 |
| Increasing social inequality  (B_3_) | 0.160 | Widening gap between rich and poor (B_31_) | 0.006 |
|  |  | Social housing inequality (B_32_) | 0.154 |
| Damage to the ecological environment  (B_4_) | 0.172 | Destruction of ecosystem (B_41_) | 0.022 |
|  |  | Disturbance of ecological balance (B_42_) | 0.009 |
|  |  | Weakening of ecological service function (B_43_) | 0.141 |
